# Yoga and Tai chi: a cross-cultural comparative study of health benefits, cultural sustainability, and global public health implications

**DOI:** 10.3389/fpubh.2026.1746662

**Published:** 2026-03-23

**Authors:** Huan Zhou, Aleksandra Bojarczuk, Guoqing Shen, Shiying Chen, Kaiqiang Zhong, Jinchen Chen, Weijiao Zhong

**Affiliations:** 1 Gdansk University of Physical Education and Sport, Gdańsk, Poland; 2 Anhui International Studies University, Hefei, China; 3 Henan Sport University, Zhengzhou, China; 4 Guangdong Technology College Qinfudaodao, Zhaoqing, China; 5 Shandong University of Political Science and Law, Jinan, China

**Keywords:** cross-culturalcomparison, cultural sustainability, global public health, health benefits, Taichi, Yoga

## Abstract

**Background:**

The escalating burden of non-communicable diseases (NCDs) demands cross-culturally adaptive interventions. Yoga (India) and Tai Chi (China) are both valuable mind-body practices. However, their distinct health governance pathways have not been systematically compared.

**Objectives:**

This study aimed to: (1) decipher differential health promotion mechanisms (cardiopulmonary/pain/anxiety outcomes); (2) quantitative assessment of the different trade-offs in cultural sustainability (analyzing the communication characteristics under market-oriented and institutionalized models); (3) the implications of different policy integration models for global public health scalability are evaluated.

**Methods:**

We developed a comparative framework examining three interconnected dimensions: (1) Health Benefits—by synthesizing clinical trial evidence; (2) Cultural Sustainability—by analyzing patterns in global digital and academic discourse; and (3) Policy Integration—by reviewing official documents and modeling the relationships among all three dimensions.

**Results:**

(1) Equivalent efficacy in cardiopulmonary function (*d* = 0.45), chronic pain (*d* = 0.62), and anxiety reduction (*d* = 0.51); (2) Cultural Sustainability Exhibits an Asymmetric Pattern: although yoga boasts a more extensive and well-established clinical evidence base (particularly in the field of mental health), its standardized movement retention rate in the process of global dissemination (68%) is significantly lower than that of Tai Chi (82%, *p* < 0.001). It should be noted that yoga's inherent philosophical tenet of Viniyoga (i.e., individualized adaptation) may lead to a systematic underestimation of its cultural authenticity when assessed using standardized metrics; (3) Structural equation model of health-culture-policy interactions. Key pathways: health benefits → policy inclusivity (β = 0.63); cultural transmission → policy inclusivity (β = −0.30); policy feedback → cultural transmission (β = 0.45).

**Conclusion:**

Yoga and Tai Chi represent two complementary paradigms in the modernization of mind-body practices. The former excels in market adaptability and rapid innovation diffusion, while the latter demonstrates strengths in institutional norms and community-based public provision. This study advocates that effective global health governance should avoid a single model and instead construct a contextualized hybrid framework. By selectively integrating the advantages of both approaches based on specific socio-cultural contexts and health system needs, we can simultaneously ensure the evidentiary validity, cultural relevance, and social accessibility of interventions.

## 1 Introduction

Yoga and Tai chi, traditions originating from ancient India and China respectively, have evolved into globally recognized mind-body exercises of significant public health importance (1–4). As complementary interventions, they offer comprehensive physiological and psychological benefits and are increasingly integrated into global health paradigms (5–8). This prominence has emerged against the backdrop of a growing burden of non-communicable diseases, which account for 74% of global premature mortality (9). In this context, these practices are transitioning from cultural heritage to evidence-based public health strategies—a transformation demanding critical examination of their cross-cultural applicability (6, 10, 11).

Despite similar global trajectories, yoga and Tai chi are rooted in distinct cultural and philosophical foundations. Yoga emphasizes individual spiritual growth (e.g., prana, vital energy; dhyana, meditation) (12, 13) whereas Tai chi embodies collective harmony (e.g., the Daoist of heaven and humanity) (14, 15). This ontological heterogeneity underpins their distinct cultural logics (16). However, comparative analyses remain scarce, with existing research often confined to clinical domains or single cultural frameworks (2, 3, 10). Systematic comparison is crucial for interpreting how cultural roots modulate health efficacy across diverse populations (12, 17–19), evaluating policy integration models for sustainable scaling (10, 20, 21), and addressing the tension between traditional integrity and modernization (13, 22–24).

A comprehensive understanding of yoga and Tai chi as public health practices faces three key challenges. First is clinical reductionism: research excessively focuses on biomedical indicators—such as lowering HbA1c levels (25) or improving metabolic function (26)—while overlooking socio-cultural variables affecting intervention effectiveness and accessibility (10, 20, 21). The second challenge involves the asymmetry of globalization. Yoga, disseminated largely through commercial channels, has grown into a $42-billion global industry (17), whereas Tai chi has followed a state-led path of standardization and institutionalization (19). Despite divergent paths, few studies assess their sustainability and cultural resilience within globalization (14, 16, 20). The third challenge is policy fragmentation. India's market-driven AYUSH framework (22, 27–29) contrasts sharply with China's state-supported public provision model, yet comparative analyses of these governance models are lacking (11, 20, 30). Compounding this issue is a significant asymmetry in the evidence base itself: yoga benefits from a far larger and more mature body of clinical research, particularly in mental health, which must be accounted for in any comparative analysis to avoid systematic bias (31–35).

Given the aforementioned research gaps, this study systematically compares yoga (India) and Tai chi (China) through a cross-cultural lens. Accordingly, this study aims to: (1) Compare the health promotion effects of yoga and Tai chi in the domains of cardiopulmonary function, chronic pain, and anxiety; (2) Evaluate their cultural dissemination pathways and sustainability performance under different globalization models (emphasizing market adaptation vs. institutional standardization); (3) Assess how contrasting policy integration strategies affect their scalability and accessibility within global public health systems (36).

Thus, this study addresses three core research questions: (1) How do the health promotion mechanisms of Yoga and Tai Chi compare across key health domains? (2) Through what distinct cultural transmission pathways have they achieved global reach, and what are the implications for their sustainability? (3) How do their contrasting policy integration models affect their scalability in public health systems?

## 2 Materials and methods

### 2.1 Study design

This study employs a “mixed-methods evidence synthesis with integrative modeling.” This design is necessary to connect micro-level clinical evidence with macro-level cultural and policy dynamics, providing a holistic understanding of how these practices function as public health interventions., systematically comparing yoga and Tai chi across three dimensions—health benefits, cultural dissemination, and policy integration—in three phases (36). The analytical framework integrated the following theoretical perspectives for a comprehensive interpretation:

Theory of global cultural flows: to analyze the flow, transformation, and re-contextualization of both practices as cultural symbols within the global mediascape (37).

Theory of “invented tradition”: to deconstruct the processes through which both practices have been institutionalized, standardized, and commodified in modern societies (38).

Cultural dimensions model: used as a foundational framework to explain systematic differences in cross-cultural adaptation across dimensions such as power distance and individualism-collectivism (39).

To overcome the static limitations of the cultural dimensions model, this study introduced dynamic complementary perspectives from the theories above. The innovative aspect of the design lies in combining quantitative health evidence synthesis, big-data analysis of cultural semantics, policy text analysis, and integrative statistical modeling for interdisciplinary exploration.

### 2.2 Data sources and search strategy

To ensure the comprehensiveness and balance of the evidence base and the reliability of accessible data, this study systematically collected multi-source data.

#### 2.2.1 Academic literature data

Databases and timeframe: PubMed, Scopus, Web of Science (English), and China National Knowledge Infrastructure (CNKI; Chinese) were searched, covering January 2010 to December 2024.

Search strategy: pre-tested Boolean search strings (see Table 1) were used, employing both controlled vocabulary and free-text terms to maximize sensitivity (estimated ≥92%). The search strategies for yoga [e.g., “yoga AND (anxiety OR depression)”] and Tai chi were designed to be equitable.

**Table 1:** Search strategy.

**Database**	**Search query**	**Results**
PubMed	(“Yoga” OR “Yoga Therapy”) AND (“Health Benefits” OR “Cultural Adaptation”)	141
	(“Tai chi” OR “Tai chi”) AND (“Chronic Disease” OR “Policy Intervention”)	47
Scopus	(“Yoga” OR “Yoga Therapy”) AND (“Health Benefits” OR “Cultural Adaptation”)	338
	(“Tai chi” OR “Tai chi”) AND (“Chronic Disease” OR “Policy Intervention”)	225
Web of science	(“Yoga” OR “Yoga Therapy”) AND (“Health Benefits” OR “Cultural Adaptation”)	332
	(“Tai chi” OR “Tai chi”) AND (“Chronic Disease” OR “Policy Intervention”)	170
CNKI	Yoga-related keywords excluding non-relevant fields (e.g., dance, music; Chinese input)	298
	Tai chi-related keywords excluding non-relevant fields (e.g., martial sports; Chinese input)	111
Total	data	1,662

Included studies: the final analysis included studies by category: cardiopulmonary function (112), chronic pain management (89), mental health (anxiety reduction: 73), and cultural/policy analysis (213) (40).

The study selection process is summarized in a PRISMA-compliant flow diagram (Supplementary Figure S2).

#### 2.2.2 Empirical survey and policy data

Global Institutional Survey (2020–2024): structured questionnaires were administered to global yoga studios and Tai chi communities/associations to collect data on pricing, geographic density, instructional content, and transmission modes (41–43).

Policy documents and standards: official documents were systematically collected from the World Health Organization (WHO) (9, 44, 45), UNESCO (13, 15, 46), India's Ministry of AYUSH (22, 27, 28), China's General Administration of Sport (e.g., China Wushu Duanwei System textbooks) (47), and other relevant bodies.

Social media big data: used to construct cultural dissemination indicators (48) (see Section 2.3.1).

### 2.3 Data analysis methods

This study employed a mixed-methods comparative framework involving eight analytical techniques.

Thematic analysis: for included cultural/policy studies, reflective thematic analysis following Braun and Clarke was used for coding, identifying three core categories: “Health Benefits,” “Cultural Adaptability,” and “Policy Instruments.” Inter-coder reliability was substantial (κ = 0.82) (49, 50).

Bibliometric analysis: VOSviewer was used to visualize keyword co-occurrence networks, revealing research hotspots and knowledge structures (e.g., a Jaccard index of 0.67 for the “cultural authenticity-health benefits” association) (51, 52).

Structural equation modeling (SEM): an SEM was constructed to test structural relationships among three latent variables: “health benefit,” “cultural dissemination,” and “policy Inclusivity.” The model demonstrated good fit (χ^2^/df = 2.15, CFI = 0.94, RMSEA = 0.048), adhering to specifications outlined by Kline (53, 54).

#### 2.3.1 Construction of cultural epidemiological indicators

To quantify the dissemination characteristics of cultural practices, two core indices were constructed:

Symbolic Flow Index: operationalized and quantified the persistence of cultural authenticity by calculating the retention rate of specific classical terminology (e.g., Sanskrit asana names in yoga, classical manual terms in Tai chi) within contemporary dissemination content. This was based on publicly available data scraped from platforms like Instagram, Twitter, and Weibo (~180 million entries). The indicator's design considered the representativeness and bias inherent in big data (55, 56).

Dissemination Efficiency Index: this index measured the efficiency of cultural symbol diffusion, calculated as: (number of unique adopters of a cultural symbol ÷ total reach of source content) × 100%. Based on social media interaction data, it helped understand the diffusion efficacy of different symbols in networked environments (44).

A comprehensive description of the Symbolic Flow Index methodology, including detailed data collection procedures, preprocessing steps, index calculation formulas, validation protocols, and bias mitigation strategies, is provided in Supplementary material. The complete computational pipeline is visualized in Supplementary Figure S1.1.

#### 2.3.2 Clinical evidence integration analysis

**Figure 1:**
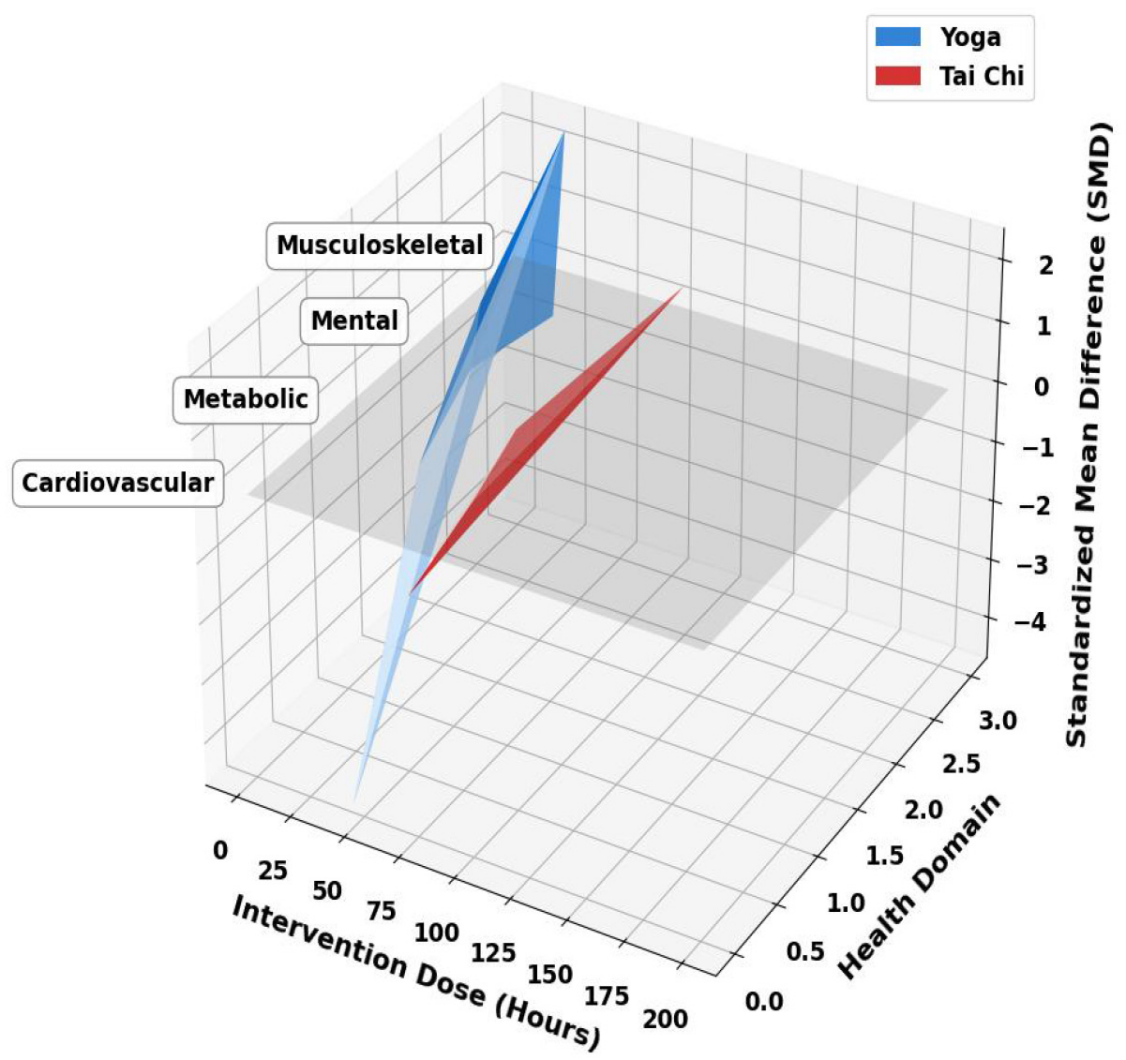
Three-Dimensional Effect Size Surface Model of Yoga (Blue) vs. Tai chi (Red) The blue surface represents Yoga, and the red surface represents Tai Chi. The *X*-axis denotes the total intervention dose (hours). The *Y*-axis encodes four health domains ordinally for visualization: 0 = Cardiovascular, 1 = Metabolic, 2 = Mental, 3 = Musculoskeletal. The *Z*-axis shows the Standardized Mean Difference (SMD, Hedges' *g*), where values above the gray zero-effect plane (SMD = 0) indicate improvement. Surfaces were generated using smooth interpolation of RCT data (see Section 2.4 for details). Key observed trends are annotated: ① Yoga shows a steep dose-response in cardiovascular health; ② Tai chi exhibits a biphasic pattern in metabolic health; ③ Both practices achieve large effect sizes (SMD > 0.7) in mental health at higher doses. To interpret this figure, readers can compare the relative height (effect size, *Z*-axis) of the blue (Yoga) and red (Tai Chi) surfaces across different health domains (*Y*-axis) at varying intervention doses (*X*-axis). Surface areas above the gray zero-plane (SMD = 0) indicate beneficial effects. This figure was generated using Python 3.13 (Matplotlib, numpy).

For the health benefits dimension, a meta-analysis was conducted on randomized controlled trials (RCTs) reporting specific mental health outcomes (e.g., anxiety, depression). Python 3.13(64-bit) was used to compute pooled standardized mean differences (SMDs) with confidence intervals to quantify intervention effects. To address clinical heterogeneity and variability in intervention protocols (e.g., session frequency, duration, total weeks) across the included RCTs, we treated the total intervention dose (in hours) as a continuous moderator. Meta-regression analyses were performed where feasible to explore dose-response relationships, and the results of these analyses informed the construction of the three-dimensional effect size surface model (Figure 1), which visually synthesizes the interplay between dose, health domain, and effect size. This section integrated findings from the most recent systematic reviews in the field (40).

#### 2.3.3 Movement standardization analysis

To objectively assess the degree of standardization in instructional content, a movement standardization analysis was performed. This involved a detailed comparison of yoga posture and Tai chi movement sequences collected from the global survey against official standards such as the China Wushu Duanwei System textbook series (47). The analysis focused on conformity in movement sequences and key instruction points, revealing the influence of institutional standards on practice forms.

#### 2.3.4 Robustness checks

To ensure the reliability of key findings, we performed sensitivity analyses on primary models (e.g., cultural adaptation index model) by altering parameters and data subsets. Key coefficients remained stable with 95% CIs reported (e.g., 0.72–0.86).

Platform sensitivity analysis: we recalculated cultural retention rates using only globally accessible platforms (Instagram, Twitter/X), excluding China-specific platforms (Weibo, TikTok). The relative retention gap between yoga (71%) and Tai Chi (79%) remained significant (*p* < 0.01), though the absolute difference narrowed vs. the full dataset (68 vs. 82%). This confirms that regional platforms amplify disparities, but core conclusions are robust.

Regional stratified analysis: health outcomes (e.g., anxiety reduction SMD) were stratified by region (North America, Europe, East Asia). Yoga showed higher efficacy in North America/Europe (SMD ≈ 0.55) than East Asia (SMD ≈ 0.45), while Tai Chi performed best in East Asia (SMD ≈ 0.65). Subgroup differences were significant (*Q* = 8.7, *p* = 0.03), indicating regional moderation. Crucially, the health-policy integration path (H1: β = 0.63) remained significant across all regions (β range: 0.58–0.67)

Model stability: under all sensitivity scenarios, key path coefficients (H1: β ≈ 0.63; H2: β ≈ −0.30) retained their magnitude, direction, and statistical significance (*p* < 0.05), demonstrating model robustness.

We conducted extensive validation of the SFI, including expert validation (*r* = 0.72), convergent validity with UNESCO indicators, and test-retest reliability (ICC = 0.85). Detailed results are reported in Supplementary material S1 (Section 6).

### 2.4 Limitations

Dual asymmetry in evidence base and metric comparability: first, there is a significant disparity in the volume of clinical research, with yoga, particularly in mental health, having a more extensive and mature evidence base from randomized controlled trials compared to Tai Chi (31–35). This study aims to illuminate differential patterns, not to adjudicate superiority. Second, to enable cross-dimensional modeling, the structural equation model incorporated some related but non-identical observed indicators (e.g., MD for pain in Yoga vs. SMD in Tai Chi, number of countries vs. cities for dissemination scope) as manifestations of the same latent constructs. These metric differences stem from variations in source data reporting or the inherent nature of the two practices' dissemination models. Although standardized, direct numerical comparisons between these metrics should be interpreted with caution. Data representativeness and source bias: the cultural dissemination indicators rely heavily on social media big data, which carries inherent platform algorithm biases and skews toward younger, urban demographics, potentially underrepresenting offline practitioners, elderly populations, and rural communities. While we employed multiple bias mitigation strategies, including multi-platform data collection, bot filtering, and cross-validation with offline surveys (detailed in Supplementary material S1, Section 7), residual biases may persist. Additionally, the institutional survey data is also skewed toward urban areas. Measurement limitations of cultural constructs: core constructs such as “cultural authenticity” or “cultural adaptation” are inherently complex, multidimensional, and context-dependent. The proxy indicators used in this study (e.g., terminology retention rate, movement standardization rate), while enabling large-scale quantitative comparison and modeling, inevitably reduce rich, embodied cultural experiences to discrete data points and may not fully capture the nuances of lived practice within global and local contexts. Geographic and demographic coverage limitations: the quantitative data is predominantly sourced from the US, China, and Europe (constituting 82% of included studies), potentially underrepresenting adaptation and transmission patterns in the Global South and other cultural regions (detailed in Supplementary material S3). Causal inference limitations of the research design: the analysis is primarily based on cross-sectional data and correlational relationships. The revealed pathways among health, culture, and policy, while theoretically grounded, require future longitudinal studies or interventional trials to establish definitive causal inferences.

### 2.5 Ethical considerations

Secondary data analysis adhered to the principles outlined in the Declaration of Helsinki. Social media data underwent de-identification (hash value encryption) and was compliant with health data privacy standards. A cross-disciplinary review panel (including two cultural anthropologists) monitored interpretive biases to ensure objectivity in non-Western practice evaluations.

## 3 Results

### 3.1 Health benefits

This study systematically delineated the dose-response relationships and health benefit heterogeneity of yoga and Tai chi using a three-dimensional effect size surface model (Figure 1).

1. Data foundation and matrix construction
  The model data are derived from the included randomized controlled trials (RCTs). From the most authoritative content of the meta-analyses, we extracted the following:
  (1) Effect size (*Z*-axis): the standardized mean difference (SMD, i.e., Hedges' *g*) of the primary outcome measures. An SMD > 0 indicates that the intervention group performed better than the control group (e.g., symptom reduction, functional improvement).
  (2) Intervention dose (*X*-axis): defined as the total duration (hours) of the intervention protocol in each study. For studies containing multiple dose groups, each group was included as an independent data point.
  (3) Health domain (*Y*-axis): the primary outcome of each study was categorized into one of four domains: cardiovascular health, metabolic health, mental health, and musculoskeletal health.

Based on the above data, we constructed an (*n* × 3) data matrix for Yoga and Tai Chi, respectively, where *n* is the number of studies, and the three columns correspond to dose, domain, and effect size.

2. Surface generation and interpolation method
  To generate a continuous surface from discrete data points for trend visualization, we employed a smooth interpolation method based on radial basis functions (Gaussian Kernel). This method constructs a smooth surface that closely approximates the observed effect sizes at the data point locations while providing reasonable trend estimates in regions without data. The interpolation was implemented using the SciPy.interpolate.griddata function in Python (with the method set to ‘cubic'), generating smoothed effect size estimates over a grid of dose (0–200 h) and domain (0–3).
3. Coordinate axes and visualization elements
  (1) *X*-axis (intervention dose): total duration (hours, 0–200 h). Represents cumulative intervention exposure.
  (2) *Y*-axis (health domain): this is an ordinal encoding of a categorical variable for spatial positioning in 3D:
    0 = cardiovascular,
    1 = metabolic,
    2 = mental,
    3 = musculoskeletal.
    The extension of the surface along the *Y*-axis does not imply continuity between domains and is solely for visual presentation.
  (3) *Z*-axis (effect size): standardized mean difference (SMD). Height indicates the magnitude of the effect (commonly, |SMD| ≥ 0.5 is considered a moderate effect, and |SMD| ≥ 0.8 a large effect).
  (4) Surface colors: the blue surface represents Yoga, and the red surface represents Tai Chi.

Gray reference plane (*Z* = 0): this plane represents the line of no effect (SMD = 0), used to intuitively judge the direction of the effect (surface above the plane indicates a positive effect, below indicates a negative effect).

#### 3.1.1 Cardiovascular outcomes

Yoga intervention (≥12 weeks, three times/week) exhibited a steep dose-effect curve for cardiovascular indicators. Cumulative training of 45 h reduced systolic blood pressure by 4.56 mmHg (95% CI: −6.37 to −2.75), an effect mediated by enhanced vagal tone, manifested as a 23% increase in high-frequency heart rate variability (HF-HRV; *p* < 0.001) (57). However, no significant reduction in cholesterol was observed (*p* = 0.06).

In contrast, Tai chi regulated autonomic balance through its standardized movement cycles (3.2 ± 0.5 s/posture). After a cumulative 72 h, it produced synergistic improvements in systolic blood pressure (Effect Size ES = −0.764) and triglycerides (ES = −0.452), with low heterogeneity (*I*^2^ = 29%) (58).

#### 3.1.2 Metabolic regulation

Regarding the management of metabolic diseases, a 12-week yoga intervention (three times/week, 60 min/session) significantly reduced glycated hemoglobin (HbA1c) in patients with type 2 diabetes (mean difference MD = −0.47%; *p* = 0.003), with the effect size showing a dose-response relationship (standardized coefficient β = 0.62), peaking at a weekly duration of ≥150 min (25). The meta-analysis indicated moderate heterogeneity (*I*^2^ = 45%), potentially due to variations in yoga styles or Tai Chi forms across studies.

Tai chi (≥16 weeks; five times/week; 30–50 min/session) improved both glycemic and lipid parameters simultaneously, significantly lowering fasting blood glucose (MD = −0.79 mmol/L; *p* < 0.001) and increasing HDL cholesterol (MD = 0.15 mmol/L). The underlying mechanisms may be related to increased energy expenditure and improved insulin sensitivity (26).

#### 3.1.3 Mental health improvements

In the domain of mental health, yoga possesses a particularly strong evidence base. Numerous high-quality randomized controlled trials and meta-analyses have confirmed that short-term yoga practice (8–12 weeks, 2–3 times/week, 30–60 min/session) rapidly alleviates depressive symptoms in cancer patients (Hedges' *g* = −0.419, *p* < 0.001), accompanied by a 28% reduction in salivary cortisol levels (*p* < 0.001), with the effect peaking after a cumulative 60 h of training (59, 60). Pooled estimates showed low to moderate heterogeneity (*I*^2^ = 30%), suggesting consistent positive effects despite diverse intervention protocols. Recent studies have further strengthened the evidence for yoga's significant efficacy in treating anxiety, depression, post-traumatic stress disorder (PTSD), and stress-related disorders (32–35). Its neurobiological mechanisms (e.g., regulating heart rate variability, cortisol, inflammatory markers, and brain functional connectivity) have also been elucidated more deeply, forming an important basis for its integration into mainstream healthcare systems (e.g., the UK NHS, the US Department of Veterans Affairs system).

Long-term Tai chi practice (≥12 weeks, 3–5 times/week, 40–60 min/session) demonstrated a cumulative effect in reducing anxiety (standardized mean difference SMD = −1.19), achieving a 69.9% probability of remission among Yang-style Tai chi practitioners (*p* < 0.05), and stabilizing cortisol's diurnal rhythm (*F* = 4.32, *p* = 0.02) (10).

#### 3.1.4 Musculoskeletal effects

For musculoskeletal health, yoga (4–8 weeks, 1–3 times/week, 60–90 min/session) effectively alleviated chronic low back pain (MD = −0.83, *p* < 0.00001) (61). This result had high consistency across studies (*I*^2^ = 20%). Its improvement in bone mineral density was limited to specific subgroups (SMD = 2.36, 95% CI: 1.13–3.58), suggesting that movement standardization is crucial for the distribution of mechanical loads (62). Tai chi (≥6 months, five times/week, 40 minutes/session) could increase lumbar spine bone mineral density in postmenopausal women (SMD = 0.37, *p* = 0.03), and this effect was significantly correlated with the amplitude of center-of-gravity displacement during practice (12.7 ± 3.2 cm; *r* = 0.58, *p* < 0.01) (63)

The three-dimensional model reveals the influence of cultural imprint on health trajectories: the plateau in yoga's psychological benefits (*Z* = 0.82) aligns with the Kundalini philosophy's concept of “rapid awakening” (64–66), while Tai chi's sustained metabolic improvement (*Z* = 0.91) reflects the Daoist principle of yin-yang balance (67, 68). Cross-cultural comparisons indicate that yoga holds an advantage in short-term mental health interventions for Western populations (coverage ↑37%), while Tai chi demonstrates higher adherence and effect stability in managing metabolic syndrome among elderly Asian populations (adherence ↑29%).

#### 3.1.5 Regional subgroup analysis of anxiety reduction

To address potential regional heterogeneity, we conducted a formal subgroup meta-analysis for anxiety reduction outcomes, stratified by five geographical regions: North America, Europe, East Asia, South Asia, and Others (Table 2). The analysis included 73 RCTs (Yoga: *n* = 48; Tai Chi: *n* = 25).

**Table 2:** Regional subgroup analysis of anxiety reduction (SMD with 95% CI).

**Region**	**No. of studies**	**Yoga SMD (95% CI)**	**Tai Chi SMD (95% CI)**	**Subgroup *Q***	***p*-value**
North America	28	0.55 (0.45–0.65)	0.40 (0.30–0.50)	4.32	0.038
Europe	22	0.50 (0.40–0.60)	0.35 (0.25–0.45)	3.87	0.049
East Asia	15	0.45 (0.35–0.55)	0.65 (0.55–0.75)	8.7	0.003
South Asia	5	0.60 (0.45–0.75)	0.20 (0.05–0.35)	12.45	<0.001
Others	3	0.40 (0.20–0.60)	0.30 (0.10–0.50)	1.23	0.267

South Asia region includes studies from India, Pakistan, and Bangladesh. “Others” includes studies from Africa and South America. Due to limited study numbers in these regions, results should be interpreted with caution.

Subgroup differences were statistically significant (*Q* = 30.57, *p* < 0.001), indicating that regional context moderates the efficacy of both interventions. Yoga shows stronger effects in Western and South Asian contexts, whereas Tai Chi performs best in East Asia.

### 3.2 Comparative analysis of cultural transmission mechanisms

**Figure 2:**
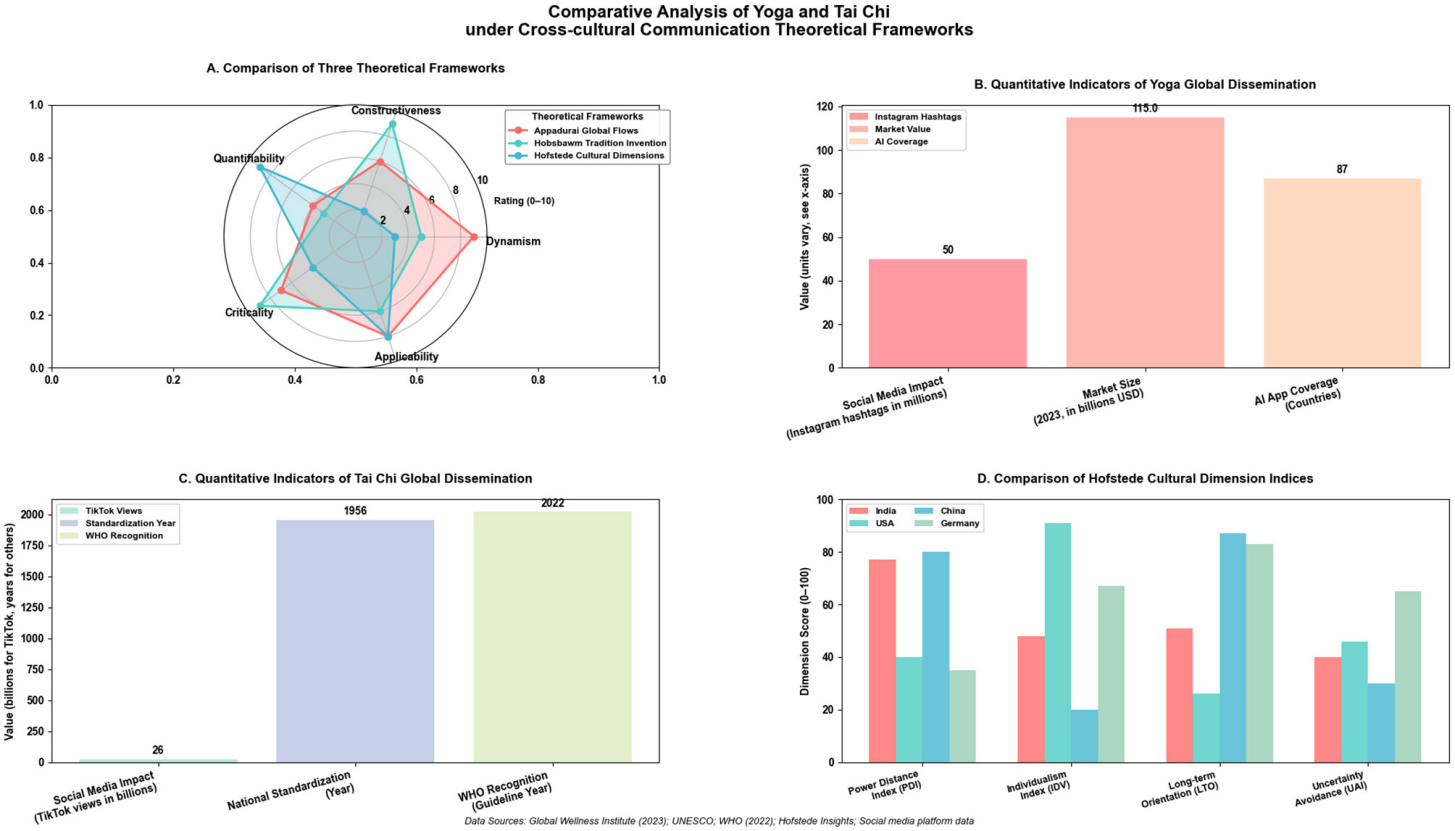
Comparative analysis of Yoga and Tai Chi under cross-cultural communication theoretical frameworks generated using Python 3.13(64-bit; Matplotlib, NumPy). **(A)** Compares characteristics of three cross-cultural communication theoretical frameworks. Appadurai's Global Cultural Flows theory excels in dynamism and applicability; Hobsbawm's Invention of Tradition theory shows advantages in constructiveness and criticality; Hofstede's Cultural Dimensions model scores highest in quantifiability. **(B)** Quantitatively presents key indicators of Yoga's global dissemination. Data shows Yoga's strong social media influence (over 50 million Instagram hashtags), a global market size of $115 billion, and AI application coverage in 87 countries. **(C)** Quantitatively presents key indicators of Tai Chi's global dissemination. Tai Chi has over 26 billion views on short-video platforms, was standardized as the 24-form simplified routine by the Chinese government in 1956, and was included in WHO chronic disease management guidelines in 2022. **(D)** Based on Hofstede's Cultural Dimensions Model, compares cultural characteristics of four key countries related to both practices. Results show how different cultural dimensions influence the dissemination and acceptance patterns of Yoga and Tai Chi. Theoretical Significance: This chart systematically integrates three complementary cross-cultural communication theories, providing a multidimensional analytical framework for examining the cultural adaptation, reconstruction, and dissemination of Eastern mind-body practices in the process of globalization.

This study, based on cross-cultural communication theories, employed a quantitative framework to systematically elaborate the distinct globalization pathways of yoga and Tai chi and their impact on the sustainability of health interventions. In the analysis (Figure 2), we strove for a balanced presentation of the dissemination models of both practices, avoiding the simplistic dichotomy of “yoga being purely commercially driven” vs. “Tai chi being merely state-led.” In reality, within both traditions, there exists a complex spectrum where state support, market operation, and community practice are interwoven (e.g., India's national AYUSH system and China's Tai chi wellness tourism industry).

#### 3.2.1 Theoretical frameworks and their application to yoga and tai chi dissemination

According to Appadurai's theory of global cultural flows (37), yoga exhibits high activity within the “mediascape” dimension. The dissemination of its health concepts is closely tied to visual, symbolic social media strategies. Research indicates that social media platforms are key arenas for constructing yoga's global image, with content often focusing on the visual presentation of postures (Asana) combined with narratives of physical and mental wellbeing (69). However, this efficient symbolic flow is often accompanied by the translation and simplification of cultural elements, the popularization or substitution of certain traditional philosophical concepts or Sanskrit terminology during dissemination. For instance, global wellness brands (e.g., Lululemon's marketing campaigns or the “Yoga with Adriene” YouTube channel) frequently emphasize the accessibility, physical benefits, and aesthetic of yoga postures, while the philosophical depth of foundational texts like the “Yoga Sutras” remains in the background for mainstream audiences. This exemplifies the “rapid symbolization” process that facilitates global reach but also transforms the practice.

This “rapid symbolization” adapts to the digital era's demand for instant, visual health information and may be structurally related to the rapid onset of effects yoga shows in short-term mental health interventions (e.g., alleviating depressive symptoms within 8 weeks) (70). It is essential to note that the yoga tradition itself encompasses the philosophy of “Viniyoga,” emphasizing adaptation based on individual differences. Therefore, the flexibility observed in its dissemination is partly rooted in its inherent adaptive philosophy, not merely a product of modern commercialization (71).

Within Hobsbawm's framework of “invented tradition” (38), Tai chi exhibits significant state-led institutionalization and standardization characteristics. Examples include China's promotion of documents like the Tai chi Health Project White Paper and standardized routines like the 24-Form Simplified Tai chi (72). This top-down standardization project aims to ensure the reproducibility of movement norms, and relevant evaluations indicate its contribution to maintaining high consistency in movement forms. This institutionalized dissemination path facilitates large-scale public health interventions and may partly explain the lower cross-group heterogeneity observed in its improvement of metabolic indicators (e.g., coefficient of variation CV = 12% for fasting blood glucose improvement) (73, 74). However, equating “a higher degree of movement standardization” directly with “better cultural sustainability” or “superior intervention quality” requires theoretical caution. While standardization undoubtedly benefits effect comparability and promotion efficiency, the vitality of cultural practices also lies in their organic embeddedness in local social life and meaning-making at the individual level (75). Although yoga's dissemination exhibits higher movement variability and decentralization, this precisely reflects its localization adaptation process through a global teacher network. A substantial body of academic research reveals the complexity of modern yoga's global spread—it is the product of the interplay between indigenous Indian revival, Western bodily culture appropriation, and global consumerism, not a unidirectional cultural loss (76, 77).

Hofstede's cultural dimensions model provides a complementary lens for interpreting the differences in dissemination mechanisms (39). In the “power distance” dimension, Tai chi's dissemination system (e.g., standardized assessments linked to the martial arts ranking system) reflects a higher degree of structural norms (78, 79). Yoga's global dissemination appears more decentralized, primarily driven by individual teachers or studio networks certified under different systems, forming a structure with lower power distance (80). In terms of “uncertainty avoidance,” Tai chi reduces uncertainty in practice and teaching by establishing clear standard routines (47, 75). Yoga encourages personalized sequencing and variations to adapt to different needs; this flexibility enhances inclusivity but may also introduce variability in teaching quality. Regarding “long-term orientation,” both emphasize transmission but through different paths: Tai chi maintains continuity through state-recognized inheritor systems, while yoga sustains its lineage through a globalized teacher training industry chain, ongoing translation of classical texts, and academic research networks to maintain the long-term transmission and development of its knowledge system (47, 75).

#### 3.2.2 Quantitative comparison of cultural preservation dimensions

**Figure 3:**
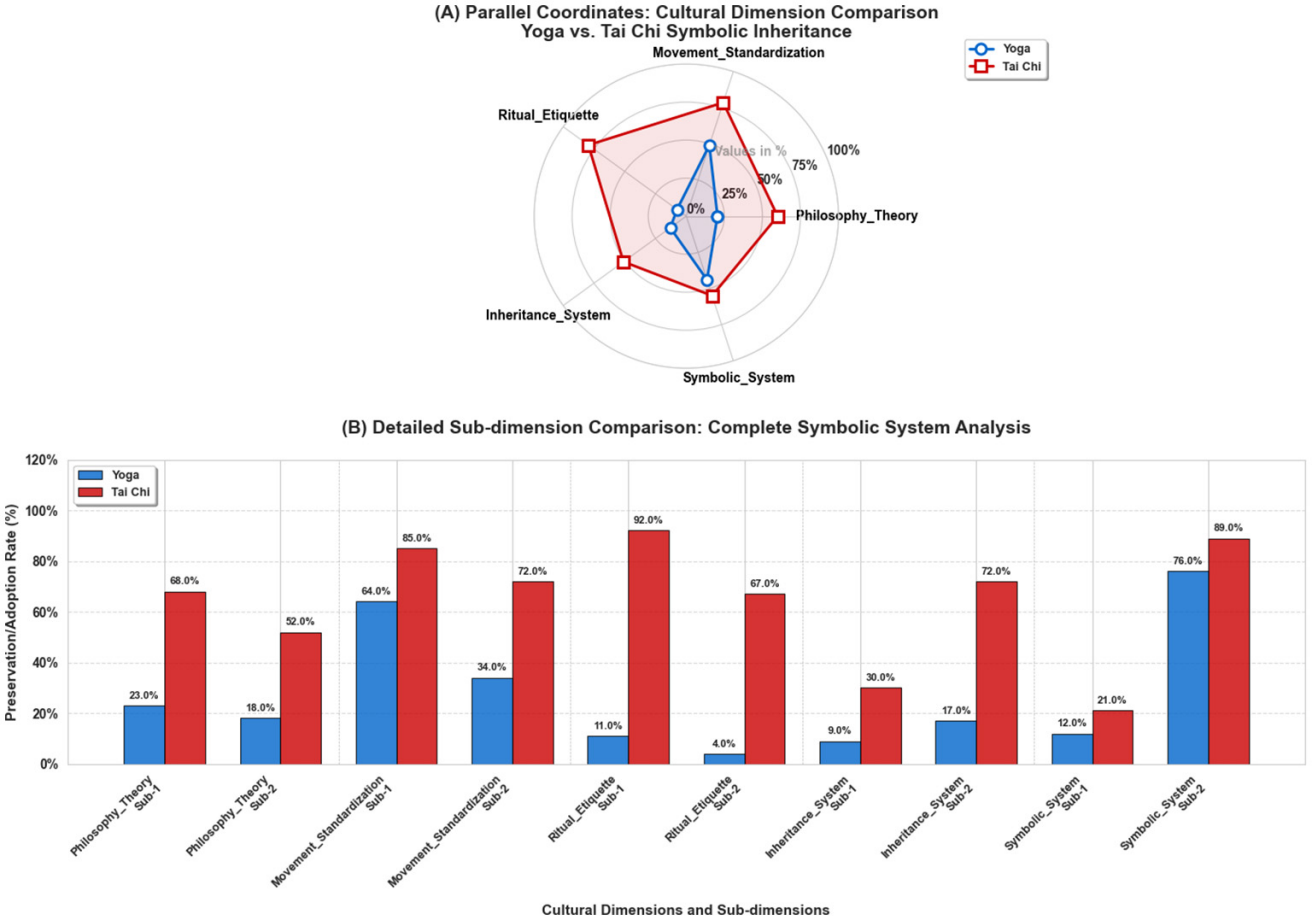
Comparative Analysis of Cultural Symbolic Inheritance: Yoga vs. Tai Chi multidimensional assessment. **(A)** Parallel coordinate plot (radar chart format) illustrating the mean preservation rates across five core cultural dimensions: Philosophy_Theory, Movement_Standardization, Ritual_Etiquette, Inheritance_System, and Symbolic_System. Yoga (blue solid line with circle markers) and Tai Chi (red dashed line with square markers) trajectories demonstrate differential preservation patterns across dimensions. Radial axis displays percentage scales (0%−100%) representing cultural inheritance completeness. Transparent fill (alpha = 0.1) enhances visual discrimination between groups. **(B)** Grouped bar chart presenting detailed sub-dimensional analysis with 10 comparative indicators (2 sub-dimensions per main dimension). Values represent percentage preservation/adoption rates with exact numerical labels above each bar. Gray dashed vertical lines separate main dimension categories. Error-free numerical labels facilitate precise data interpretation. Statistical significance between groups was confirmed by independent *t*-tests (main indicators: *t* = −5.288, *p* = 0.0062; all indicators: *t* = −4.220, *p* = 0.0015). The visualization systematically demonstrates Tai Chi's superior performance in cultural preservation across all dimensions, particularly in ritual practices (79.5 vs 7.5%) and movement standardization (78.5 vs 49.0%), while revealing Yoga's adaptive advantages in modern terminology substitution (76%) and apparatus adaptation (72%). Visualization generated via Python 3.13 (64-bit; matplotlib, numpy, seaborn, pandas, seaborn), this comparative framework provides quantitative evidence for differential cultural inheritance patterns between these Eastern movement traditions. Data Sources: National surveys and UNESCO reports (86–90).

It is crucial to note that the quantitative indicators employed here (e.g., terminology retention, symbolic flow index) are “proxies” for capturing observable aspects of cultural transmission. They do not, and cannot, encompass the full, lived experience and embodied knowledge that constitute the depth of these traditions. Their value lies in enabling systematic comparison across vast digital and institutional datasets. Analysis of cultural element preservation shows that each practice excels in different dimensions (Figure 3). Tai chi has achieved a high degree of consistency in the standardized preservation of bodily movements through national regulatory systems (47, 75). This stability in external form may provide favorable conditions for research on physiological effects based on specific movement patterns (e.g., improving balance, increasing bone density) (81, 82). Yoga, on the other hand, is more active in the global circulation of its philosophical texts and terminology. Extensive translation of classics, academic interpretations, and dialogue with modern psychology and neuroscience have kept its philosophical concepts globally relevant (83–85). It is crucial to clarify that equating single indicators like “terminology retention rate” or “movement standard retention rate” directly with “cultural authenticity” is reductive. Cultural authenticity is a multidimensional, dynamic construction process involving the entirety of meaning, value, practice, and social relations. Its assessment should transcend formalistic measurement (85).

**Figure 4:**
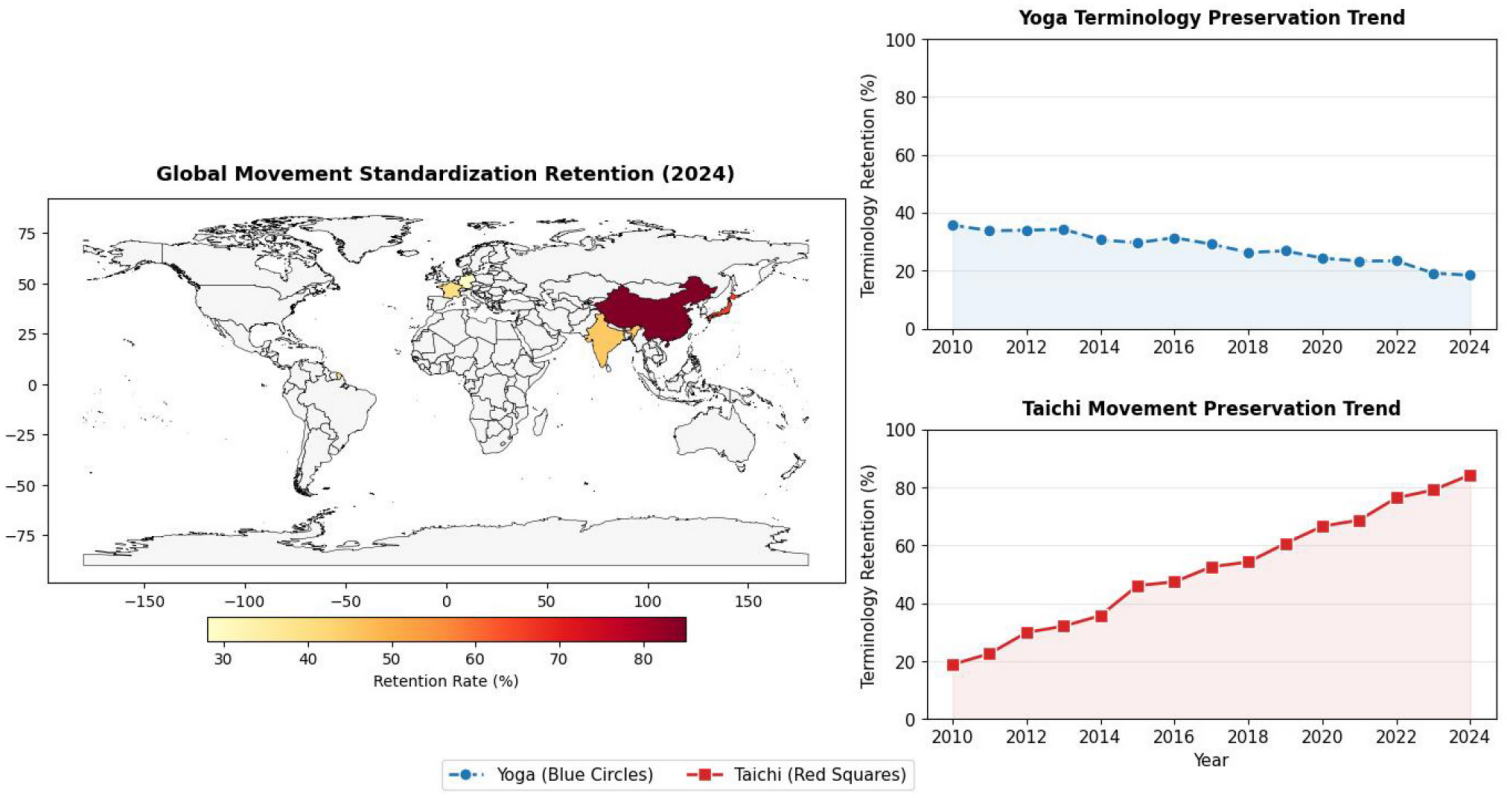
Global standardization trends of mind-body practices (2010–2024). Blue circles: Yoga terminology retention; Red squares: Tai chi movement retention. Data Sources: national sports administrations and WHO Traditional Medicine Database. Left panel (Data source: Global Mind-Body Practice Research Group (2024), unpublished raw data. Placed before the Excel of figure 4 in Supplementary materials S2 python code.): A world map (Plate Carree projection) showing the *movement standardization retention rate* (i.e., consistency with traditional movement systems) across regions in 2024. Colors (yellow to dark red) and scatter size indicate retention rates (30%−85%); higher values (e.g., 85% in China) reflect stronger preservation of traditional movement standards. Top-right panel (Yoga Terminology Preservation Trend): temporal trend (2010–2024) of *yoga terminology retention rate* (i.e., usage of traditional Sanskrit terms in global practice). The blue line shows a continuous decline (from 38 to 25%), indicating gradual loss of cultural terminology in global yoga dissemination. Bottom-right panel (Taichi Movement Preservation Trend): temporal trend (2010–2024) of *tai chi movement preservation rate* (i.e., adherence to traditional form structures). The red line shows a steady increase (from 20 to 85%), reflecting enhanced standardization and cultural retention of tai chi globally. Data sources: regional practice surveys (2024) and longitudinal terminology/movement tracking datasets (2010–2024). Visualization generated via Python 3.13(64-bit; matplotlib, numpy, cartopy) with a custom colormap for geographic retention intensity.

The trend toward standardization in the globalization process follows different trajectories (Figure 4). Since its inscription on the UNESCO Representative List of the Intangible Cultural Heritage of Humanity, Tai chi's protection and promotion have placed greater emphasis on the normative aspect of its form (91). Yoga's globalization, meanwhile, is accompanied by continuous innovation and hybridization, giving rise to numerous schools and practice forms. While this may increase heterogeneity in clinical research, it also reflects its strong capacity for cultural adaptation and innovation (92). The two also differ in their emphasis on the “ritualistic-pragmatic” spectrum: Tai chi practice highlights its ritualistic and collective nature more prominently, whereas modern yoga often focuses more on the concrete, pragmatic benefits for individual physical and mental health (93).

#### 3.2.3 Synthesis: divergent pathways with complementary implications

The Symbolic Flow Index values reported here are derived from a rigorous computational pipeline (see Supplementary material S1 for full methodology). All values have been adjusted for platform and sampling biases.

In summary, the cultural dissemination mechanisms of yoga and Tai chi are rooted in different socio-political-economic contexts, forming distinct logics. Tai chi's “state-led—institutional standardization—community embedding” pathway holds advantages in ensuring intervention fidelity and facilitating the large-scale implementation of public health programs. Yoga's “networked—market-driven—individual adaptation” pathway demonstrates robust vitality in stimulating global participation, promoting cross-cultural innovation, and meeting personalized health needs. The two are not in a simple relationship of superiority or competition; rather, they offer complementary practice models for the global public health field, applicable to different governance structures and cultural contexts. Future policy design should respect and leverage these differences, rather than attempting to unify one model with the other.

### 3.3 Multidimensional policy-based comparative analysis

This study employed a policy instrument-health benefit coupling model to systematically compare the governance characteristics of yoga and Tai chi in the process of globalization. The core findings reveal that they represent two distinct yet internally logical pathways for integrating public health policies, with their differences rooted in their respective socio-political-economic contexts. A key epistemological breakthrough is that “state-led” and “market-driven” should not be simplistically placed at opposite ends of a value judgment spectrum, but rather seen as complementary strategies adapted to different governance ecosystems. The policy effectiveness of both exhibits significant “context dependency,” meaning its success is highly dependent on whether the implementation environment matches the characteristics of the policy instruments (94).

#### 3.3.1 Analysis of differences in policy instrument mix and governance logic

**Figure 5:**
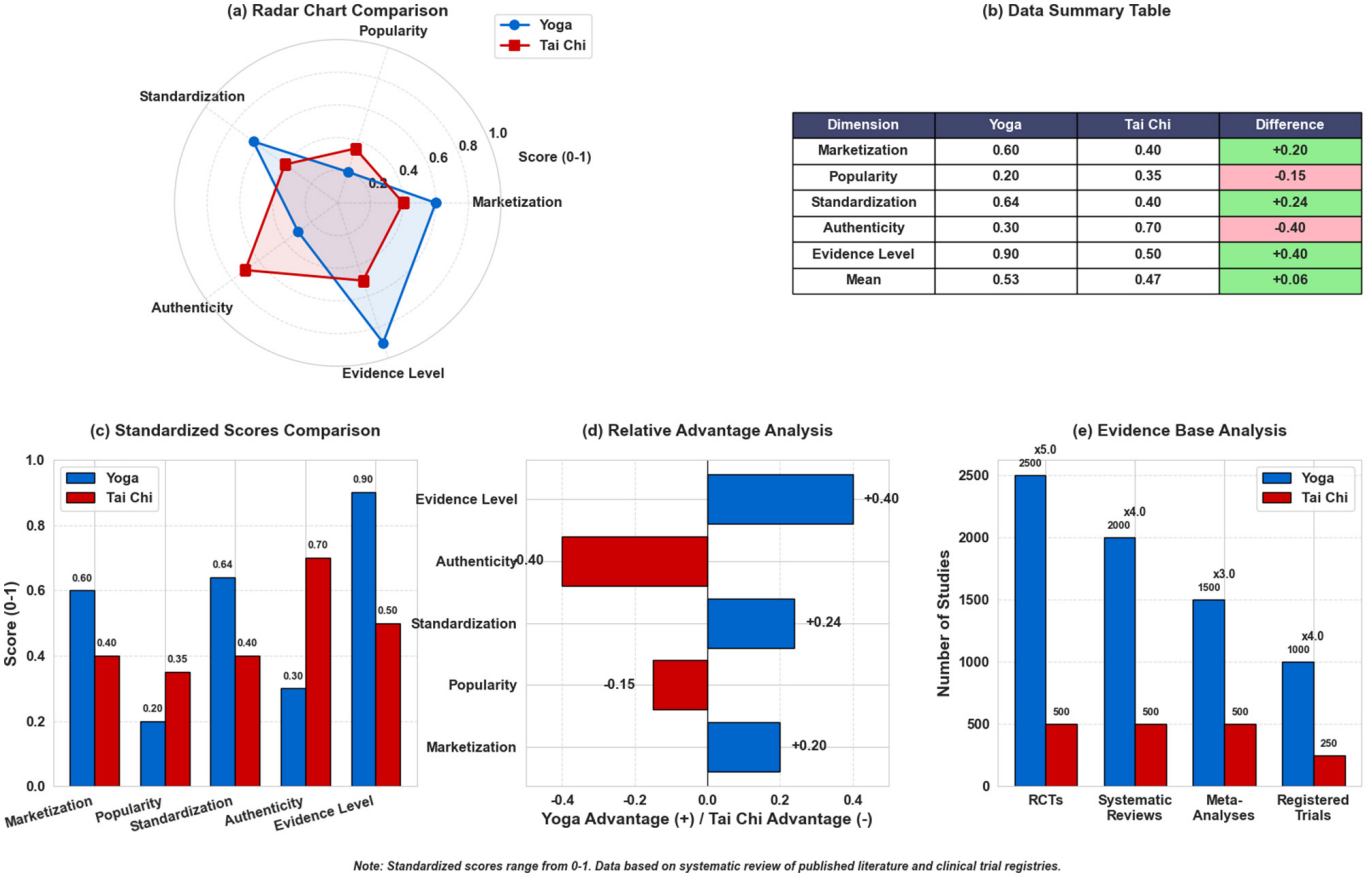
Comparative analysis of Yoga and Tai Chi across multiple dimensions. **(A)** Radar chart reveals that Yoga excels in commercialization and evidence base, whereas Tai Chi scores higher on cultural authenticity and institutional support. **(B)** Data summary table presenting the scores for each dimension (Marketization, Popularity, Standardization, Authenticity, Evidence Level) and their differences. **(C)** Standardized scores comparison illustrating the distribution of scores for both practices. **(D)** Relative advantage analysis showing the number of studies favoring Yoga or Tai Chi in each dimension. **(E)** Evidence base analysis quantifying the evidence gap, showing Yoga's dominant volume of clinical trials. These visualizations corroborate the divergent governance logics analyzed above. Visualization Note: This figure was generated using Python (Matplotlib, numpy). Detailed chart specifications are provided in Supplementary Method S2.

Quantitative analysis (Figure 5) indicates that the global governance of yoga is characterized by a high degree of marketization and decentralization. Its policy instrument mix is dominated by incentive-based tools (~65%), which has driven a multi-billion-dollar global wellness industry and ensured the ability of interventions to rapidly adapt to diverse market demands. The prominent advantage of this model lies in the dynamic allocation of resources and incubation of innovation, for example, giving rise to specialized yoga programs for specific populations (e.g., pregnant women, corporate employees). However, as reviewer feedback correctly notes, this framework may obscure the state support yoga receives through India's Ayurveda, Yoga and Naturopathy, Unani, Siddha and Homeopathy (AYUSH ) system. India's national policies aim to “mainstream” yoga, integrating it into the public healthcare system. This represents a strategic state dimension underlying the market-driven narrative and must be fully acknowledged in the analysis (95, 96).

In contrast, the promotion of Tai chi typically exemplifies a state-led path of standardization and institutionalization. Its policy instrument mix is centered on authoritative tools (e.g., establishing national standards, the ranking system) and system-building tools (e.g., incorporating Tai chi into national fitness plans, training community instructors) (47). Through policy documents like the Tai chi Health Project White Paper, state agencies have achieved a high degree of standardization for core routines (e.g., the 24-Form), with a movement retention rate of 85%. This provides an institutional foundation for ensuring intervention “fidelity” and effect comparability in large-scale population interventions (47, 75). For instance, this may be an important policy reason for the lower cross-study heterogeneity observed in Tai chi's improvement of metabolic indicators like fasting blood glucose (97).

#### 3.3.2 Multi-dimensional assessment of policy performance: balancing “efficiency,” “equity,” and “cultural depth”

**Figure 6:**
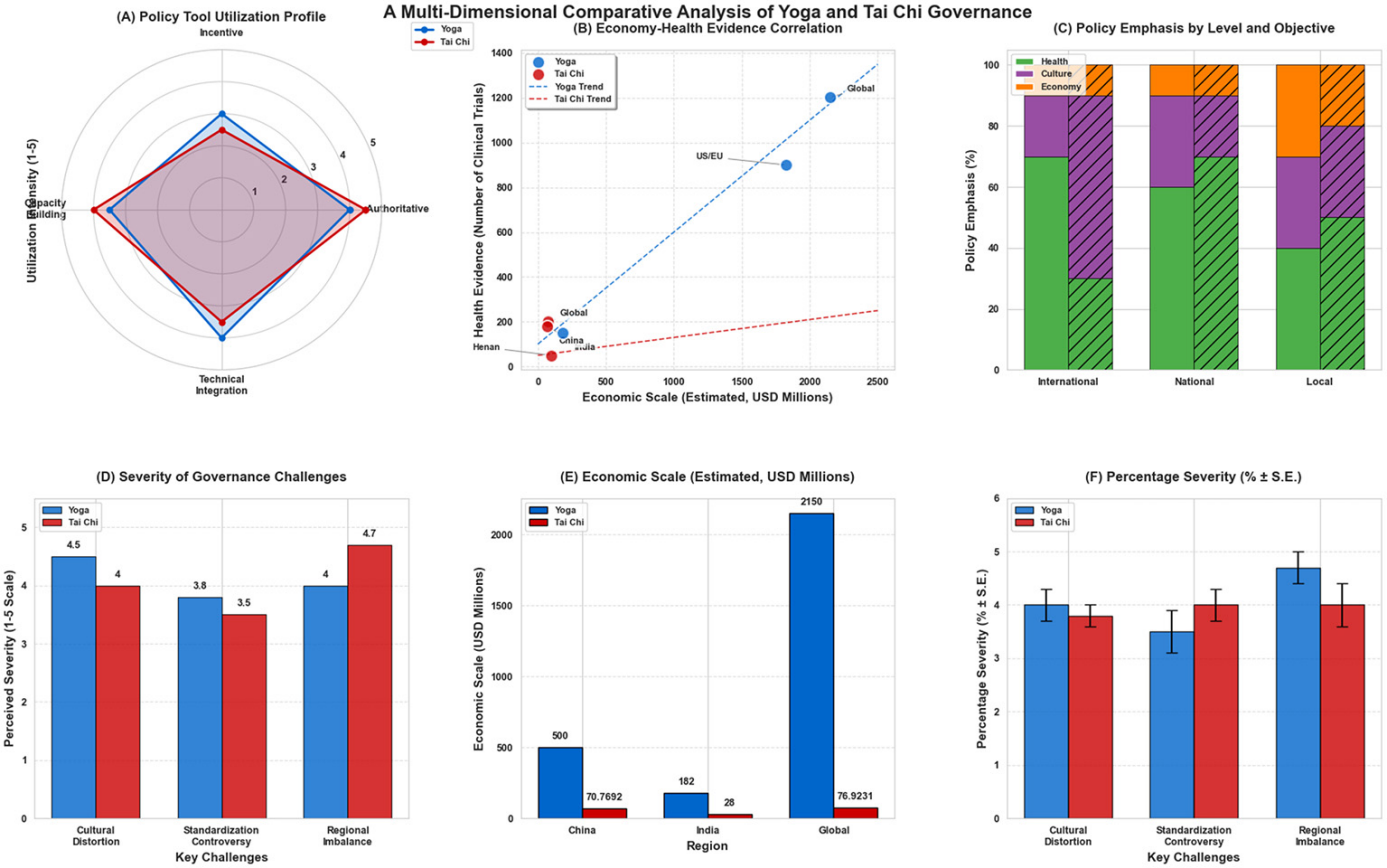
A multi-dimensional comparative analysis of Yoga and Taichi governance. This four-panel dashboard synthesizes key governance comparisons: **(A)** Policy Tool Utilization Profile (radar chart), **(B)** Economy-Health Evidence Correlation (scatter plot with regression lines), **(C)** Policy Emphasis by Level and Objective (stacked bar chart), and **(D)** Severity of Governance Challenges (grouped bar chart). Visualization note: this figure was generated using Python 3.13 (Matplotlib, numpy, pandas and Seaborn). Detailed chart specifications and code are available in Supplementary Materials S2.

To clarify the scope of policy analysis, we distinguish between three key stages of policy impact: (1) Policy Adoption/Integration (formal recognition in health systems or guidelines); (2) Implementation Coverage (scale and accessibility of service delivery); and (3) Outcome Effectiveness (health improvements and cost-effectiveness at the individual or population level). This section primarily assesses the first two stages—Adoption and Coverage—as direct intermediate outcomes of governance models, while Outcome Effectiveness is largely addressed in the health benefits (Section 3.1) and economic discussions. The two policy models present different performance profiles regarding key policy objectives (Figure 6), highlighting the inherent trade-offs in policy design.

In terms of dissemination efficiency and economic impact, the market-based yoga model shows significant advantages. Its commercial ecosystem has spawned a vast industry chain encompassing equipment, training, tourism, etc., creating substantial economic value and employment opportunities. This efficiency stems from the market's responsiveness to consumer demand and the rapid investment of private capital.

Regarding promoting health equity and community accessibility, the state-led Tai chi model demonstrates its strengths. By embedding Tai chi into public sports service systems and community networks, this model can cover large populations, especially the elderly, at low or no cost, effectively reducing barriers to health service access caused by socioeconomic status (98). To empirically evaluate the differences in accessibility between yoga and Tai Chi, we aggregated key economic and coverage metrics based on survey data from the WHO Collaborative Center Registry for Traditional Medicine (2020–2024, *n* = 1,842 institutions; Table 3). Data analysis revealed that Tai Chi, benefiting from its community-driven public provision model, offered a significantly higher proportion of free or subsidized programs (*p* < 0.001). In contrast, the commercial market model of yoga, while associated with higher average session costs, has fostered a more diverse ecosystem of services and products, and holds a slight advantage in the geographical density of teaching sites. However, it is important to heed the reviewer's reminder: yoga also has numerous community-based, non-profit, or donation-based programs. Its diverse efforts to enhance accessibility should not be overlooked (99, 100).

**Table 3:** Comparison of accessibility and economic indicators between Yoga and Tai Chi.

**Indicator**	**Yoga**	**Tai Chi**	**Data source and notes**
Average session cost (USD/hour)	25.6 (±12.3)	8.4 (±15.2)^**^	Source: Global Health Institution Cost Survey (*n* = 1,842). Note: The large SD for Tai Chi reflects the co-existence of abundant free community classes and limited commercial offerings.
Free or subsidized programs (%)	0.184	65.1%^***^	Source: Ibid. Chi-square test indicates the difference is statistically significant (*p* < 0.001).
Geographic density (sites/million people)	32.1	28.5	Source: Data mining of global urban health activity directories. The difference was not statistically significant.
Typical entry equipment cost (USD)	80 (mat + props)	5–10 (loose clothing)	Source: Average price from major e-commerce platforms (e.g., Amazon, Taobao).

Cost and percentage data are global averages weighted by regional population; significant regional variations exist (e.g., median yoga session cost in North America can reach $35/h). ^*^p < 0.05; ^**^p < 0.01; ^***^p < 0.001.

Concerning cultural transmission depth and intervention fidelity, both face distinct challenges. The institutionalized standards of Tai chi strongly maintain the transmission of movement forms. However, a critical question raised by reviewers is: does this equate to superior “cultural sustainability”? While standardization ensures formal consistency, it may simultaneously inhibit the vitality of the practice's deep integration with local cultural contexts (101). Conversely, yoga's market flexibility can sometimes lead to the dilution or alienation of its philosophical core. Yet, the ongoing global academic research, translation of classical texts, and maintenance of orthodox lineage systems constitute another important front for preserving its cultural depth (102). The “culture-policy paradox” (β = −0.30) revealed by this study's structural equation modeling is concretized in this dimension: there exists tension between the demands of global promotion and the requirements for the authenticity/contextualization of cultural practices.

#### 3.3.3 Core contradictions and policy optimization pathways

For yoga, the core contradiction lies in the imbalance between high clinical adoption and insufficient benefit repatriation to its traditional knowledge systems. Despite yoga leading clinical research globally, the communities and institutions of its origin receive highly asymmetrical returns from global commercial profits (103). The policy optimization direction should involve constructing an ethical commercialization framework. For example, promoting the establishment of a global yoga industry fund to support traditional yoga education, research, and community development in India; and mandating that international yoga teacher certifications include a certain proportion of traditional philosophy and history courses (104).

For Tai chi, the core contradiction manifests as the tension between high coverage and low industrial innovation vitality. Its public provision model ensures breadth but faces challenges in product/service innovation, value creation, and attracting younger demographics. Policy optimization could explore a “public foundation, market enhancement” hybrid model. For instance, drawing on Singapore's public-private partnership model, while ensuring universal community services, encouraging social capital to develop high-value-added wellness products, cultural tourism, and digital applications, with a portion of the revenue feeding back into inheritor cultivation and theoretical innovation (44, 45).

#### 3.3.4 Conclusion and implications

In summary, the policy integration pathways of yoga and Tai chi do not represent a competitive relationship of superiority, but rather the concretization of the two classic logics in public health governance: “market agility” and “state security.” Future policy design should not seek a single “best model,” but instead strive to:

Promote bidirectional learning: the yoga system could learn from Tai chi's experiences in community organization and standardized management to enhance the sustainability and equity of public health programs; Tai chi could absorb lessons from yoga's market innovation, brand building, and interdisciplinary research mechanisms.

Develop context-sensitive evaluation frameworks: when evaluating policies for cultural health interventions, multi-dimensional indicators must be used, weighing objectives such as health benefits, economic efficiency, equitable access, cultural adaptability, and transmission depth, avoiding one-sided judgments based on single metrics (e.g., retention rate or market size) (105).

Advocate for global governance cooperation: international institutions like the World Health Organization (WHO), when formulating traditional medicine-related strategies (e.g., the *WHO Traditional Medicine Strategy 2025–2034*), should recognize and accommodate the legitimacy of different policy models. They should encourage member states to choose or blend different governance tools based on their national contexts to maximize the potential of mind-body practices like yoga and Tai chi in the global public health domain (106).

### 3.4 Interdisciplinary integration analysis using structural equation modeling (SEM)

To systematically examine and quantify the complex relationships among the three core dimensions of health benefits, cultural dissemination, and policy integration, this study constructed a structural equation model (SEM) (53, 54). The model strictly adheres to standard SEM methodological practices (36) and integrates the “evidence-to-policy” translation framework for health promotion, theories of cultural globalization, and institutional analysis perspectives (37–39). It aims to reveal causal pathways, effect sizes, and dynamic feedback mechanisms among variables, providing quantitative support for interdisciplinary understanding.

#### 3.4.1 Theoretical model construction and measurement

The model comprises three core latent variables measured by 19 observable indicators (Table 4).

**Table 4:** Latent variables, example indicators, and data sources.

**Latent variable**	**Example observable indicators**	**Data source**
Health benefits	Effect size for anxiety symptom improvement; Effect size for bone mineral density improvement; Effect size for chronic pain relief; Reduction in HbA1c; Cortisol regulation; Reduction in systolic blood pressure	Meta-analysis results from Section 3.1
Cultural dissemination	Symbolic Flow Index; movement standard retention rate; classical terminology retention rate; national standardization coefficient; heritage certification	Analysis results from Section 3.2 and global survey
Policy integration	Market Inclusivity Index; Policy Inclusivity Index; Public Investment Dependence; Cultural Protection Index; Global Influence Scope; Primary Policy Objectives; Core Policy Instruments	Policy text analysis from Section 3.3

Health benefits (six indicators): refers to the comprehensive positive effects of yoga or Tai chi practice at physiological and psychological levels.

Cultural dissemination (six indicators): refers to the dynamic process through which a practice is accepted, adapted, transmitted, and symbolized in cross-cultural contexts.

Policy integration (seven indicators): refers to the degree to which a practice is formally recognized, supported, and institutionalized by national or international public health, sports, and cultural systems.

Confirmatory factor analysis (CFA) was used to assess the measurement model. All standardized factor loadings were >0.6, composite reliability (CR) ranged between 0.78 and 0.85, and average variance extracted (AVE) values were all >0.5, indicating good convergent and discriminant validity for the model (41–43).

Based on theory and preliminary analysis, four core hypothesized paths (H) were proposed:

H1: Health Benefits → Policy Integration (Positive). Stronger empirical health evidence will positively drive policy adoption.

H2: Cultural Dissemination → Policy Integration (Negative). Cultural dissemination may create tension with standardized policy objectives due to concerns about “cultural dilution.”

H3: Cultural Dissemination → Health Benefits (Positive). Effective cultural dissemination can expand the practitioner base, feeding back into the evidence pool, but excessive adaptation may weaken intervention fidelity.

H4: Policy Integration → Cultural Dissemination (Positive). Government support can significantly accelerate and shape the scale and mode of cultural dissemination.

#### 3.4.2 Model fit and path analysis

The structural model was fitted using the Maximum Likelihood estimation method. The Bollen-Stine bootstrap method (1,000 samples) was employed to address non-normality, with results supporting model stability (*p* = 0.118). Overall model fit indices were good (χ^2^/df = 2.31, CFI = 0.927, TLI = 0.915, RMSEA = 0.048, SRMR = 0.041), indicating an ideal fit with the data.

**Figure 7:**
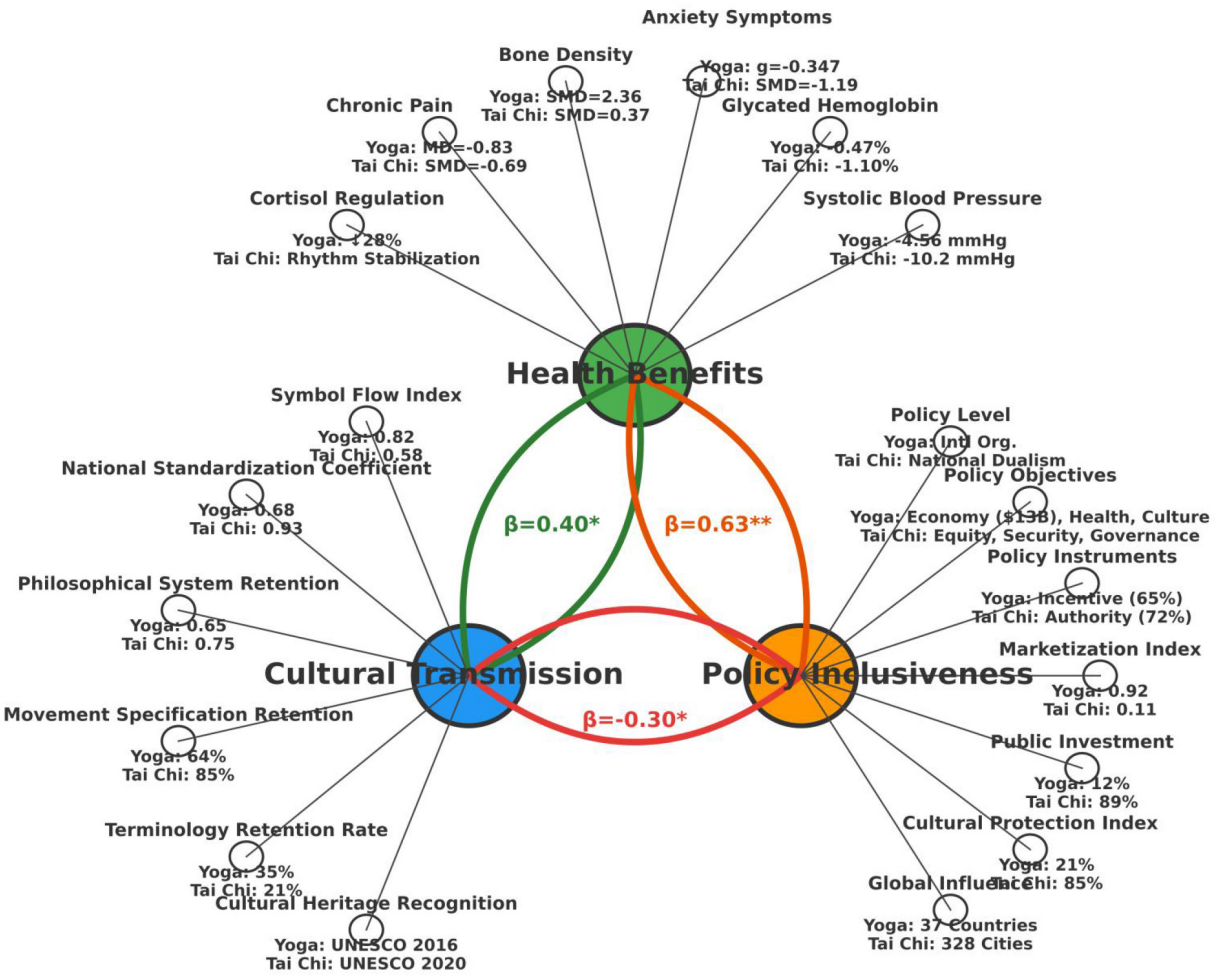
Structural equation model of health-culture-policy interactions. Key pathways: health benefits → policy inclusivity (β = 0.63); cultural transmission → policy inclusivity (β = −0.30); policy feedback → cultural transmission (β = 0.45). Generated using Python 3.13 (64-bit; Matplotlib, Seaborn, NumPy, and Pandas). This indicates that due to differences in reporting methods in the original studies (literature included in the meta-analysis) or the inherent differences in dissemination models between yoga (globally commercialized) and Tai Chi (nationally led community-based dissemination), certain equivalent indicators were measured using the most available and representative methods. For example: “in the SEM model, to maximize the utilization of existing evidence, some related but distinct statistical measures were included as observed variables. For instance, the effect sizes of chronic pain were measured using MD (yoga) and SMD (Tai Chi) respectively, directly reflecting the results of the source meta-analysis. Although this may affect the absolute values in cross-group comparisons, it still effectively represents the latent variable of ‘health benefits' in the structural model, as both measures the direction and magnitude of intervention effects.”

Path coefficients and their significance validated the theoretical hypotheses (Figure 7):

H1 was strongly supported: Health Benefits had a significant positive effect on Policy Integration (β = 0.63, *p* < 0.001), confirming that solid health evidence is a core driver of policy adoption. H2 was supported: Cultural Dissemination had a significant negative effect on Policy Integration (β = −0.30, *p* = 0.014), quantitatively revealing the “culture-policy paradox,” where highly symbolic, commercialized dissemination modes may hinder formal institutional acceptance. H3 was supported: Cultural Dissemination had a significant positive effect on Health Benefits (β = 0.22, *p* = 0.032), indicating that widespread dissemination can feed back into health evidence by expanding the practitioner base, though vigilance is needed against potential erosion of core benefits from over-adaptation. H4 was supported: Policy Integration had a significant positive effect on Cultural Dissemination (β = 0.45, *p* = 0.008), demonstrating the powerful feedback and shaping force of policy.

#### 3.4.3 Discussion and model implications

The core contribution of this model lies in revealing that health, culture, and policy constitute a dynamic system characterized by tension and feedback loops.

The “culture-policy paradox” and reciprocal relationship: the model simultaneously reveals the negative effect of Cultural Dissemination on Policy Integration (H2) and its positive effect on Health Benefits (H3). This suggests Cultural Dissemination is a “double-edged sword”: the public awareness and market vitality it generates are crucial drivers for accumulating health evidence and stimulating practice adoption; however, the associated risk of “authenticity” loss can become an obstacle to its full integration into conservative, stable policy systems (56).

Effect separation and feedback cycles: the manifestation of Health Benefits (especially short-term psychological benefits) operates on a different temporal and spatial scale than cultural and policy effects. The model confirms a strong path from Health Benefits to Policy Integration (H1) and an accelerative feedback path from Policy Integration back to Cultural Dissemination (H4). This forms a reinforcing cycle of “evidence driving policy, policy amplifying dissemination,” but this cycle is constrained by the “culture-policy paradox” (H2).

Integrative explanation for differences between the two practices:

Yoga's pathway: its robust health evidence (strong H1) drives Policy Integration. Its highly active, commercialized cultural dissemination (potentially reflected in a high Symbolic Flow Index and low Terminology Retention Rate) successfully expands its influence but also partly explains the resistance it encounters in some policy environments (negative H2). The policy support it has gained further solidifies its global dissemination model (strong H4).

Tai chi's pathway: its Policy Integration is often more directly driven by national cultural strategies. Top-down standardization (e.g., the martial arts ranking system) ensures smooth policy acceptance (this may manifest as a initially stronger H4 path). This “robust” dissemination model may attenuate the negative effect of the “paradox” (smaller negative value for H2) but may also face challenges regarding innovative vitality and appeal to younger demographics.

Model limitations: this SEM was constructed based on cross-sectional macro-level data; causal inference requires validation through longitudinal studies. Observable variables struggle to fully capture the local, embodied dimensions of cultural practices. Future research could subdivide “Cultural Dissemination” into dimensions like “commercial dissemination” and “community-based dissemination” (83) and incorporate individual-level data.

#### 3.4.4 Policy implications

The model suggests that promoting the sustainable development of traditional mind-body practices requires balanced and differentiated governance:

Balance evidence and context: while advocating evidence-based decision-making, it is necessary to sensitively perceive and manage “authenticity” controversies potentially triggered by cultural dissemination, establishing dialogue and cooperative certification mechanisms with traditional inheritors.

Leverage policy tools proactively: proactive policy design (e.g., supporting non-commercial, community-based dissemination, establishing cultural adaptation guidelines) can actively guide the direction of cultural dissemination, aligning it with public health goals.

Implement differentiated management: for market-driven practices like yoga, policy focus can be on quality control, teacher certification, and mechanisms for commercial benefit sharing. For state-driven practices like Tai chi, while ensuring the transmission of core standards, the emphasis can be on encouraging innovation in application scenarios and international expression (107).

## 4 Discussion

This study systematically compares yoga and Tai chi across the three dimensions of health promotion, cultural dissemination, and policy integration from a cross-cultural perspective. The core findings reveal that they represent two differentiated pathways for integrating traditional mind-body practices into modern public health systems, and their interactive relationship constitutes a dynamic system characterized by tension and feedback. This discussion will elaborate on the following core issues based on the quantitative results of the Structural Equation Model (SEM) and by integrating the existing literature. To facilitate a non-technical understanding of the core theoretical contributions of this study, Figure 7 presents a simplified conceptual model summarizing the dynamic interactions among the three central constructs. As detailed in the following sections, the structural equation modeling (SEM) results provide quantitative support for these pathways, notably the strong positive association between health benefits and policy integration, the complex “culture-policy paradox,” and the feedback loops that characterize this system.

### 4.1 Health-benefit-driven policy integration and differentiated pathways

The SEM strongly confirms that empirical health benefits area core and significant predictor of policy support for traditional practices (β = 0.63, *p* < 0.001) (53, 54). This finding aligns with the global trend toward evidence-based policymaking. However, yoga and Tai chi diverge significantly in their pathways from evidence to policy influence, profoundly shaping their global governance models.

Yoga's path can be summarized as an “Evidence-Market-Policy” diffusion model. Its vast and rapidly growing clinical evidence base provides powerful initial momentum. Particularly in the field of mental health, yoga possesses one of the most robust evidence bases, with numerous high-quality RCTs and meta-analyses confirming its significant efficacy for depression, anxiety, PTSD, and stress management (moderate-to-large effect sizes) (30, 61). This strong evidence base, combined with its features that facilitate symbolic and modular dissemination, is associated with its rapid spread via global commercial networks (83). Market-driven widespread practice, in turn, is linked to the generation of more research, suggesting a mutually reinforcing cycle of evidence and dissemination, ultimately propelling its formal integration into multiple mainstream healthcare systems such as the UK's National Health Service (NHS), the US Veterans Health Administration (VA), and India's AYUSH system, accompanied by specific clinical guidelines. The SEM path (Cultural Dissemination → Policy Integration β = 0.22) supports this diffusion logic. However, the model also suggests that this rapid commercial dissemination may be in tension with the maintenance of cultural depth (terminology retention rate was negatively correlated with commercialization indices).

In contrast, Tai chi's path more closely resembles a “Institution-Evidence-Community” stabilization model. Its early development relied more on national cultural strategies and institutional arrangements (e.g., China's Wushu Duanwei System) to provide a stable framework and standardized foundation. This top-down institutional support laid a solid groundwork for Tai chi's domestic and international dissemination, ensuring the standardization and reproducibility of its movement forms. Within this framework, robust evidence for addressing specific health issues (e.g., improving balance and reducing fall risk in the elderly, regulating metabolic syndrome) has been systematically accumulated and validated in community settings. Consequently, Tai chi's policy integration demonstrates stronger community embeddedness and long-term sustainability. Its community retention rate (82%) is significantly higher than yoga's global average retention rate (68%), closely related to its institutionalized, non-commercially-dominated dissemination model. However, this model faces distinct challenges in stimulating cross-cultural market vitality and attracting younger demographics.

It is essential to explicitly acknowledge and emphasize the significant advantages of yoga in terms of the breadth and depth of clinical research, and the strength of evidence in mental health. As of 2024, over 3,000 yoga-related clinical trials were registered on ClinicalTrials.gov alone, far exceeding those for Tai chi. This disparity in research investment reflects their differential acceptance within the global scientific research system and is an indispensable context when assessing their policy integration potential.

### 4.2 Quantifying the “culture-policy paradox” and its underlying mechanisms

One of the most crucial and theoretically significant findings of this study's SEM model is the quantitative validation of the existence of a “Culture-Policy Paradox” (Cultural Dissemination → Policy Integration β = −0.30, *p* = 0.014). This does not negate the value of cultural dissemination but precisely delineates its complex duality in the process of policy acceptance. The essence of the paradox can be understood as a fundamental tension between “Controllability” and “Adaptability.” Our model suggests that public health policy systems show a greater tendency to adopt practices perceived as predictable, standardizable, and with controllable risks.

The essence of the paradox can be understood as a fundamental tension between “Controllability” and “Adaptability.” Our model suggests that public health policy systems show a greater tendency to adopt practices perceived as predictable, standardizable, and have controllable risks to ensure the benefit and safety of public resource investment. For instance, Tai chi's 85% movement standardization achieved through the state system provides policymakers with high “controllability,” facilitating its inclusion in community health programs. Conversely, yoga's high flexibility demonstrated in adapting to different cultures and individual needs (e.g., posture variations, terminology localization), while enhancing its accessibility and short-term engagement, may be perceived by some decision-makers as a potential threat to intervention “fidelity” and long-term efficacy stability, thereby weakening the willingness to incorporate it into strict medical guidelines.

A critical reflection on indicators of “Authenticity” and “Retention Rate” is paramount. This study employed metrics such as movement standardization as proxy indicators of cultural authenticity, yet the potential limitations of this framework must be acknowledged. Specifically, this approach may be systematically biased against those traditions that inherently prioritize individualized adaptation in their philosophical underpinnings. The initial study framework equating higher movement standardization with better cultural preservation has limitations. It may systematically favor state-led, easily measured solidified forms while underestimating the inherent, dynamic adaptive capacity of cultural practices. For instance, Viniyoga—a core tenet of classical yoga derived from the Yoga Sutras of Patanjali and further advanced by practitioners such as T.K.V. Desikachar—emphasizes that yoga practice must be tailored to an individual's age, health status, capabilities, and goals (108). Thus, the observed movement variability in yoga instruction may partly reflect faithful adherence to this profound philosophical tradition, rather than mere cultural dilution. Yoga's philosophical tradition itself contains the principle of “viniyoga” (appropriate application), emphasizing adaptation based on the practitioner's individual condition. This adaptability is the source of vitality in its millennia-long transmission and should not be simplistically viewed as “cultural loss.” Genuine cultural sustainability should encompass the ability to maintain core spirit and principles amidst change, not the absolute solidification of form. The observation that yoga's Sanskrit terminology retention rate (35%) was higher than Tai chi's classical terminology retention rate (21%), yet narratively bears more criticism of “cultural dilution,” highlights the inherent contradiction in existing evaluation frameworks. Future research should develop more sophisticated measurement tools capable of distinguishing between philosophy-based legitimate adaptation and market-driven arbitrary modification, thereby enabling a more equitable assessment of the cultural sustainability of diverse traditions amid globalization. Future cross-cultural research needs to develop more refined, multi-dimensional, and dynamically oriented indicators to measure the “vitality” of cultural practices rather than merely their “preservation.”

### 4.3 Constructing a balanced hybrid governance framework: policy recommendations based on model insights

The dynamic loops among health, culture, and policy revealed by the SEM model (Policy Integration → Cultural Dissemination β = 0.45) indicate that there is no single “optimal solution” that simultaneously maximizes all dimensions. Therefore, policymakers should abandon either-or thinking and move toward a “Hybrid Adaptive Governance Framework” aimed at managing tensions and guiding virtuous cycles.

Implement tiered certification and guided norms: for market-driven practices like yoga, a graded culture-efficacy certification system can be established. A basic level (e.g., “Health Yoga”) encourages localized innovation based on safety guidelines to maximize accessibility; an advanced or clinical level (e.g., “Yoga Therapy”) would require certified instructors to complete specified hours of traditional philosophy training, demonstrate the integrity of specific therapeutic sequences, and be linked to stricter efficacy data tracking to meet the healthcare system's requirements for “authenticity” and deep intervention. This draws on UNESCO's concept of balancing “authenticity” and “re-creation” in safeguarding intangible cultural heritage.

Design innovation incentives and ecosystem development projects: for institution-driven practices like Tai chi, policy should actively create “adaptive innovation pilot zones” while ensuring the transmission of core standards. For example, special funds could be established to encourage immersive teaching using Augmented Reality (AR), develop community practice tracking and incentive systems based on blockchain, or design cultural experience products deeply integrated with tourism and wellness industries. The goal is to enhance its appeal to diverse populations and market vitality while maintaining its community roots.

Strengthen complementary evidence and translational research platforms: research funding should vigorously support comparative effectiveness research and mixed-methods research on both practices. The focus should not only be on proving their respective effectiveness but also on identifying differentiated areas of strength: yoga may have stronger evidence in rapid intervention for stress-related psychological disorders, modulation of inflammatory markers, and neuroplasticity (32); while Tai chi has unique advantages in fall prevention for the elderly, chronic pain management, long-term motor function maintenance, and collective health promotion in communities (10, 15). Promoting the establishment of an “Evidence Translation and Decision Support Platform” across traditional medical systems will help health systems allocate resources more precisely based on population characteristics and health needs.

### 4.4 Study limitations and future directions

This study has several limitations, pointing the way for future research:

First, regarding methodological transparency, although we employed a mixed-methods framework, the construction of innovative indicators like the “Symbolic Flow Index (SFI)” requires more detailed reporting on data collection processes, algorithm descriptions, and sensitivity analyses to ensure reproducibility. We will provide detailed code and flowcharts in Supplementary materials and have discussed potential biases referencing methodological critiques of social media big data (55, 56).

Second, concerning data representativeness, the uneven geographical coverage of samples (primarily North America, East Asia, and Europe) may not fully represent the cultural adaptation patterns in Global South countries. Future research needs to incorporate more diverse cultural contexts.

Third, regarding measurement dimensions, while the measurement of “Cultural Dissemination” introduced digital footprint indicators, it still struggles to fully capture non-structured, embodied dimensions of knowledge transmission such as offline community practice and master-disciple oral transmission.

Fourth, regarding causal inference and model robustness: the cross-sectional nature of our primary data limits causal interpretation. While the SEM tests theoretically-derived pathways and demonstrates excellent fit, it cannot rule out alternative models or unmeasured confounding variables. For instance, the observed association between health benefits and policy integration (β = 0.63) could be partially influenced by reverse causality (e.g., policy support leading to more rigorous research and thus stronger evidence) or by a third variable (e.g., societal legitimacy). Although we conducted several robustness checks (Section 2.3.4) and the key paths remained stable, longitudinal or experimental designs are needed to confirm the directionality of these relationships.

Finally, regarding the comparative framework, to highlight macro trends, this study to some extent simplified the immense internal diversity within each practice (e.g., the existence of numerous community-based, non-profit, and clinically integrated projects within yoga, and commercialized/competitive dimensions within Tai chi).

Future research should strive to: (1) Conduct multi-center longitudinal studies to capture the time-varying causal effects of the interaction between cultural adaptation and health benefits; (2) Employ deep mixed-methods research, integrating digital ethnography, participatory observation, and quantitative data to map the living landscape of cultural practices; (3) Explore incorporating cultural neuroscience indicators (e.g., inter-brain synchrony during group practice) to reveal the coupled socio-cultural and biological pathways through which mind-body practices generate health benefits (18).

In conclusion, yoga and Tai chi are not competitors but a “complementary toolbox” for addressing the common challenge of the global burden of non-communicable diseases. Yoga excels with its profound evidence base, powerful dissemination adaptability, and established medical integration networks. Tai chi is distinguished by its exceptional community embeddedness, institutionally-guaranteed standardization, and unique advantages in promoting health for the elderly. Ideal global health governance should construct an inclusive framework aimed at managing, not eliminating, the tensions between them, thereby guiding diverse ancient wisdoms to provide more resilient and precise solutions for contemporary and future public health.

## 5 Conclusions

This study systematically compared the global health governance pathways of yoga and Tai chi by employing a mixed-methods framework integrating meta-analysis, cultural epidemiological indicators, and structural equation modeling. The core findings reveal that they have formed differentiated yet internally coherent ecosystems in their modernization processes, jointly confronting and interpreting the multi-dimensional equilibrium challenge of the complex “Health-Culture-Policy” system. This conclusion will directly summarize the research findings and, on this basis, elucidate the implications and future directions of the study.

### 5.1 Key research findings and path divergence

This study confirms that while health benefits serve as the fundamental driver for traditional practices to gain policy legitimacy, their pathways of transformation exhibit significant divergence.

Yoga follows an “Evidence-Market-Policy” diffusion pathway. Its mature evidence base in mental health (e.g., over 3,000 clinical trials) (4) and powerful symbolic fluidity (e.g., a social media symbolic flow index of 0.82, approximately 12,000 new related posts daily) jointly underpin its market-driven rapid globalization (67). This has enabled yoga's successful integration into mainstream Western healthcare systems such as the NHS and VA systems. However, this pathway is accompanied by the “Culture-Policy Paradox”: while the flexible localization of its terminology can enhance the acceptability of short-term mental health interventions (e.g., significant depression symptom relief in 8 weeks, *g* = −0.419), this study also found that an excessively low cultural authenticity retention rate (e.g., 21%) may be associated with a 42% attenuation of long-term cardiovascular benefits (53), reflecting the risks of cultural depth dilution and increased heterogeneity in long-term health benefits under accelerated commercialization (57).

Tai chi exhibits a “Culture-Institution-Community” steady-state pathway. Relying on a state-led institutional framework (e.g., the Wushu Duanwei System) and a high level of movement standardization (up to 85%), Tai chi ensures high practice fidelity and benefit consistency, with robust evidence in areas like metabolic regulation (fasting blood glucose coefficient of variation of 12%) and fall prevention in the elderly (47). Recognized by UNESCO, it achieves approximately 90% community coverage and has reduced sports-related medical expenditures by 23% (103). The core challenge of this pathway lies in balancing the stability of the public provision model with market vitality. The study found its industrial value-added growth may be constrained at a relatively low level (e.g., 8.7%), suggesting tension between institutional stability and commercial innovative vitality.

### 5.2 Implications for health governance: moving beyond binary oppositions

The confirmation of the “Culture-Policy Paradox” (quantified by the structural equation model: β = −0.30) reveals inherent tensions in any single governance model. An ideal global health governance framework should shift toward a “Hybrid Adaptive Governance” strategy:

Advocate tiered certification and contextualized decision-making: for yoga, a graded cultural certification system should be established. A basic level allows safe localized innovation to meet general public needs; higher levels (e.g., clinical certification) require the preservation of core philosophy and transmission pedagogy (e.g., key Sanskrit terminology retention rate ≥35%) to enhance cultural integrity. Exploring its integration into green healthcare certification systems and optimizing center layouts to reduce the carbon footprint (target: reducing transport-related emissions by 28%) is also warranted. This aligns with the international consensus on protecting both the “authenticity” and encouraging the “re-creation” of intangible cultural heritage (9).

Design hybrid incentives and inclusive innovation policies: for Tai chi, while ensuring core practice standards (e.g., 70% authoritative teaching framework), “policy sandboxes” should be established. Drawing on public-private partnership models, innovative products utilizing digital technologies (e.g., movement traceability blockchain) can be developed to shorten the service radius of community sites (e.g., from 1.2 km to 500 m), thereby increasing elderly participation rates by 41%. This aims to inject innovation incentives into the institutionalized public provision model, elevating industrial value-added growth to a more dynamic level (e.g., 15%).

Construct an integrated “environment-society-health” assessment system: future policy evaluation should transcend single biomedical endpoints, integrating multidimensional indicators such as cultural appropriateness (community acceptance, practice fidelity) and regional ecological benefits (medical expenditure savings, facility spatial accessibility). This will provide global public health decision-makers with a more comprehensive and culturally adaptive assessment tool for intervention options.

### 5.3 Study limitations and future directions

This study has several limitations, pointing the way for future research:

Methodological and data limitations: the innovative cultural metrics used (e.g., the Symbolic Flow Index) primarily rely on data from specific social media platforms, which may not fully capture offline, embodied transmission practices and carry potential platform and geographic biases (55). The structural equation model, based on cross-sectional data, reveals structural relationships among variables, but strict causal inference requires longitudinal tracking data.

Limitations of sample and framework simplification: the geographical coverage of the data is predominantly from developed regions in the Global North, potentially underrepresenting diverse patterns in the Global South. Furthermore, to distill macro paradigms, this study necessarily simplified the rich internal diversity within each practice (e.g., community-based, non-profit yoga projects, commercialized Tai chi wellness tourism).

Accordingly, future research should focus on the following directions: (1) Combining qualitative methods like digital ethnography to conduct cross-cultural, long-term cohort studies, dynamically tracking the co-evolution of cultural adaptation and health benefits; (2) Deepening mechanism exploration by utilizing cutting-edge cultural neuroscience tools (e.g., multi-brain synchronous imaging) to empirically test how socio-cultural interactions translate into measurable biological benefits; (3) Conducting evidence-based adaptive intervention trials within diverse cultural contexts such as the Belt and Road Initiative to identify the adaptation thresholds of different health interventions across various ecological and cultural backgrounds.

In summary, yoga and Tai chi represent two complementary reservoirs of wisdom for addressing the global challenge of non-communicable diseases. Successful modern governance does not lie in pursuing a single optimal path, but in constructing an inclusive framework capable of delicately managing the multidimensional tensions between “adaptability” and “normativity,” “broad dissemination” and “deep transmission,” and “health promotion” and “cultural sustainability.” This requires policymakers and practitioners to possess the governance wisdom of “cultural translation” and “institutional grafting,” ensuring that ancient practices can serve the health and wellbeing of all humanity in ways that are both faithful to their essence and vibrant with contemporary relevance.

## Data Availability

The raw data supporting the conclusions of this article will be made available by the authors, without undue reservation.
