# Supplementary material for: Yoga and Tai chi: a cross-cultural comparative study of health benefits, cultural sustainability, and global public health implications
Source: Front Public Health. 2026 Mar 23;14:1746662. doi: 10.3389/fpubh.2026.1746662 (PMC13051707; doi:10.3389/fpubh.2026.1746662)
Supplement: Supplementary file 1 [file Data_Sheet_1.docx]

This document supplements the main article: 'Yoga and Tai chi: A Cross-Cultural Comparative Study of Health Benefits, Cultural Sustainability, and Global Public Health Implications.' All methods described here correspond to the analysis presented in Section 2.3.1 and results in Section 3.2 of the main text.

Supplementary Materials S: Detailed Methodology of Symbolic Flow Index (SFI)

1. Overview

The Symbolic Flow Index (SFI) quantifies the cultural authenticity preservation in the global dissemination of Yoga and Tai Chi by analyzing social media discourse. This document provides comprehensive methodological details to ensure transparency and reproducibility.

2. Data Collection Pipeline

2.1 Platforms and APIs

**Primary Platforms:** Instagram (Graph API), Twitter/X (Academic API v2), Weibo (Open API), TikTok (Research API), YouTube (Data API v3)

**Secondary Sources:** Reddit (Pushshift), Pinterest (API)

**Data Access Period:** January 2010 - December 2024

**Ethical Compliance:** All data collection complied with platform terms of service

2.2 Search Strategy and Keywords

**Table S1.1: Search Keywords by Platform and Language**

| **Platform** | **Language** | **Yoga Keywords** | **Tai Chi Keywords** |
| --- | --- | --- | --- |
| All | English | "yoga", "asana", "pranayama", "yoga practice", "yoga benefits", "yoga therapy" | "taiji", "tai chi", "taijiquan", "qigong", "chi flow" |
| Weibo/TikTok | Chinese | "瑜伽", "瑜伽体式", "瑜伽练习", "瑜伽治疗" | "太极拳", "太极养生", "太极功夫", "气功" |

2.3 Geographic Filtering Protocol

**Primary Method:** User profile location metadata

**Secondary Method:** Post geotags when available

**Tertiary Method:** Text-based location inference using Named Entity Recognition (NER)

**Region Classification:** North America, Europe, East Asia, South Asia, Latin America, Africa, Oceania

2.4 Sampling Framework

**Total Collected Posts:** 182,457,392 (2010-2024)

**Stratified Sampling:** Proportional to platform market share and regional internet penetration

**Temporal Distribution:** Equal monthly sampling across 15-year period

3. Data Processing and Cleaning

3.1 Deduplication Algorithm

**Python code**

# Pseudocode for deduplication

def deduplicate_posts(posts):

seen_hashes = set()

unique_posts = []

for post in posts:

content_hash = hash(post['text'] + post['image_hash'])

if content_hash not in seen_hashes:

seen_hashes.add(content_hash)

unique_posts.append(post)

return unique_posts

3.2 Language Filtering

**Languages Retained:** English, Chinese (Simplified and Traditional)

**Tools Used:** langdetect Python library (accuracy: 99.2%)

**Manual Validation: 1**0,000 randomly selected posts validated by human coders (κ = 0.91)

3.3 Spam and Bot Detection

**Three-Layer Filtering System:**

**Rule-based:** Remove posts with >10 hashtags, commercial URLs, repetitive content

**ML Classification:** Random Forest classifier trained on 50,000 labeled posts

Precision: 0.94, Recall: 0.89, F1-score: 0.91

**Manual Review:** 1% random sample reviewed by research assistants

3.4 Geographic Tagging Enhancement

**BERT-based Location Classifier:** Fine-tuned on 100,000 geotagged posts

**Confidence Threshold:** 0.85 for automated tagging

**Manual Coding:** All low-confidence posts reviewed by coders (ICC = 0.83)

4. Feature Extraction Methodology

4.1 Terminology Retention Rate (Rᵢ)

**Traditional Terminology Database:**

Yoga: 250 Sanskrit terms from Patanjali's Yoga Sutras and Hatha Yoga Pradipika

Tai Chi: 180 classical Chinese terms from Taijiquan Classics and traditional manuals

Calculation Formula: $R_{i}=\frac{\sum_{t=1}^{T} \mathbb{I}(term_{t}\in post_{i})}{T}\times100\%$

where $T$ is the total number of traditional terms for each practice.

**BERT Model Specification:**

**Base Model**: bert-base-multilingual-cased

**Fine-tuning:** 50,000 annotated social media posts

**Accuracy:** 93.7% on test set

4.2 Interaction Weight (wᵢ)

Composite Engagement Score:$w_{i}=\log_{10} (1+L_{i}+2\times S_{i}+0.5\times C_{i})$

where:

$L_{i}$​= number of likes

$S_{i}$​= number of shares/retweets

$C_{i}$​= number of comments

Platform Normalization: Z-score normalization within each platform

5. Symbolic Flow Index (SFI) Computation

5.1 Mathematical Formulation

$${SFI}_{r,y}=\frac{\sum_{i=1}^{N_{r,y}} w_{i}^{*}\times R_{i}^{*}}{\sum_{i=1}^{N_{r,y}} w_{i}^{*}}$$

$w_{i}^{*}$​= standardized interaction weight (z-score by platform)

$R_{i}^{*}$​= standardized terminology retention rate (z-score by platform and region)

$N_{r,y}$ = number of posts in region $r$ and year $y$

5.2 Aggregation Steps

**Monthly Aggregation:** Calculate SFI for each month-region combination

**Annual Averaging:** 12-month rolling average to reduce seasonal effects

**Regional Pooling:** Weighted average by population and internet penetration

6. Validation Framework

6.1 Expert Validation Protocol

**Expert Panel:** 3 cultural anthropologists (10+ years experience each)

**Rating Scale:** 1-10 for cultural authenticity

**Correlation Analysis:** Pearson's r = 0.72 (95% CI: 0.68-0.76)

**Inter-rater Reliability:** ICC(3,k) = 0.84

6.2 Convergent Validity Assessment

Table S1.2: Correlation with Established Measures

| **Measure** | **Correlation with SFI** | **p-value** |
| --- | --- | --- |
| UNESCO ICH Indicators | 0.65 | <0.001 |
| Academic Publication Count | 0.58 | <0.001 |
| Traditional Teacher Density | 0.71 | <0.001 |

6.3 Reliability Analysis

**Test-Retest Reliability:** ICC = 0.85 (6-month interval)

**Split-Half Reliability:** Spearman-Brown coefficient = 0.88

**Internal Consistency:** Cronbach's α = 0.79 across sub-indices

7. Bias Assessment and Mitigation

7.1 Platform Bias Analysis

Table S1.3: Platform Demographics vs. General Population

| **Platform** | **Age Bias** | **Gender Bias** | **Urban Bias** |
| --- | --- | --- | --- |
| Instagram | Younger (Δ = -8.2 years) | Female (65%) | High (Δ = +22%) |
| Weibo | Middle-aged (Δ = +3.1 years) | Balanced | Very high (Δ = +35%) |
| Twitter | Older (Δ = +5.4 years) | Male (62%) | Moderate (Δ = +15%) |

Mitigation Strategy: Post-stratification weighting using national census data

7.2 Sampling Bias Correction

**Offline Practitioner Adjustment:** Survey of 5,000 practitioners used to calibrate online-offline ratio

**Elderly Population Inclusion:** Partnership with 50 senior centers for offline data collection

**Global South Representation:** Oversampling in underrepresented regions (Africa, South Asia)

7.3 Content Production Bias

Correction Factors Applied:$\mathrm{CF}=\frac{GeneralPractitionerRate}{ActivePosterRate}$

Based on survey data showing only 28% of practitioners regularly post on social media.

8. Supplementary Figures

Figure S1.1: SFI Calculation Flowchart

Figure S1.1 provides a visual overview of the complete Symbolic Flow Index calculation pipeline, from data acquisition through validation and analysis.

Figure S1.2: Data Collection Timeline and Volume

Figure S1.2 shows the monthly and annual data collection volume from 2010 to 2024. The upper panel displays monthly variations with key events annotated, while the lower panel presents annual totals. The total collection comprises approximately 180 million posts, with an average of 1.0 million posts per month.

Figure S1.3: Geographic Distribution of Collected Data

Figure S1.3 illustrates the global distribution of collected social media data. Point density indicates regional data intensity, while labels show the total posts (in millions) for each region. North America and Europe represent the largest data sources, followed by East Asia and South Asia.

9. Data Collection Statistics

Table S1.4: Regional Data Collection Summary (2010-2024)

| **Table S1.4 Regional Data Colle** | | |
| --- | --- | --- |
| **Region** | **Posts (M)** | **% of Total** |
| North America | 45.2 | 25 |
| Europe | 38.7 | 21.4 |
| East Asia | 32.5 | 18 |
| South Asia | 25.8 | 14.3 |
| Southeast Asia | 12.3 | 6.8 |
| Latin America | 9.6 | 5.3 |
| Oceania | 8.4 | 4.6 |
| Africa | 5.2 | 2.9 |
| Middle East | 3.3 | 1.8 |
| Total | 181 | 100 |

Note: Platform distribution varies by region based on local market dominance and API accessibility.
