# Supplementary figures and images for: Yoga and Tai chi: a cross-cultural comparative study of health benefits, cultural sustainability, and global public health implications
Source: Front Public Health. 2026 Mar 23;14:1746662. doi: 10.3389/fpubh.2026.1746662 (PMC13051707; doi:10.3389/fpubh.2026.1746662)

Supplementary Figure S1. Flowchart of Symbolic Flow Index (SFI) Calculation Pipeline

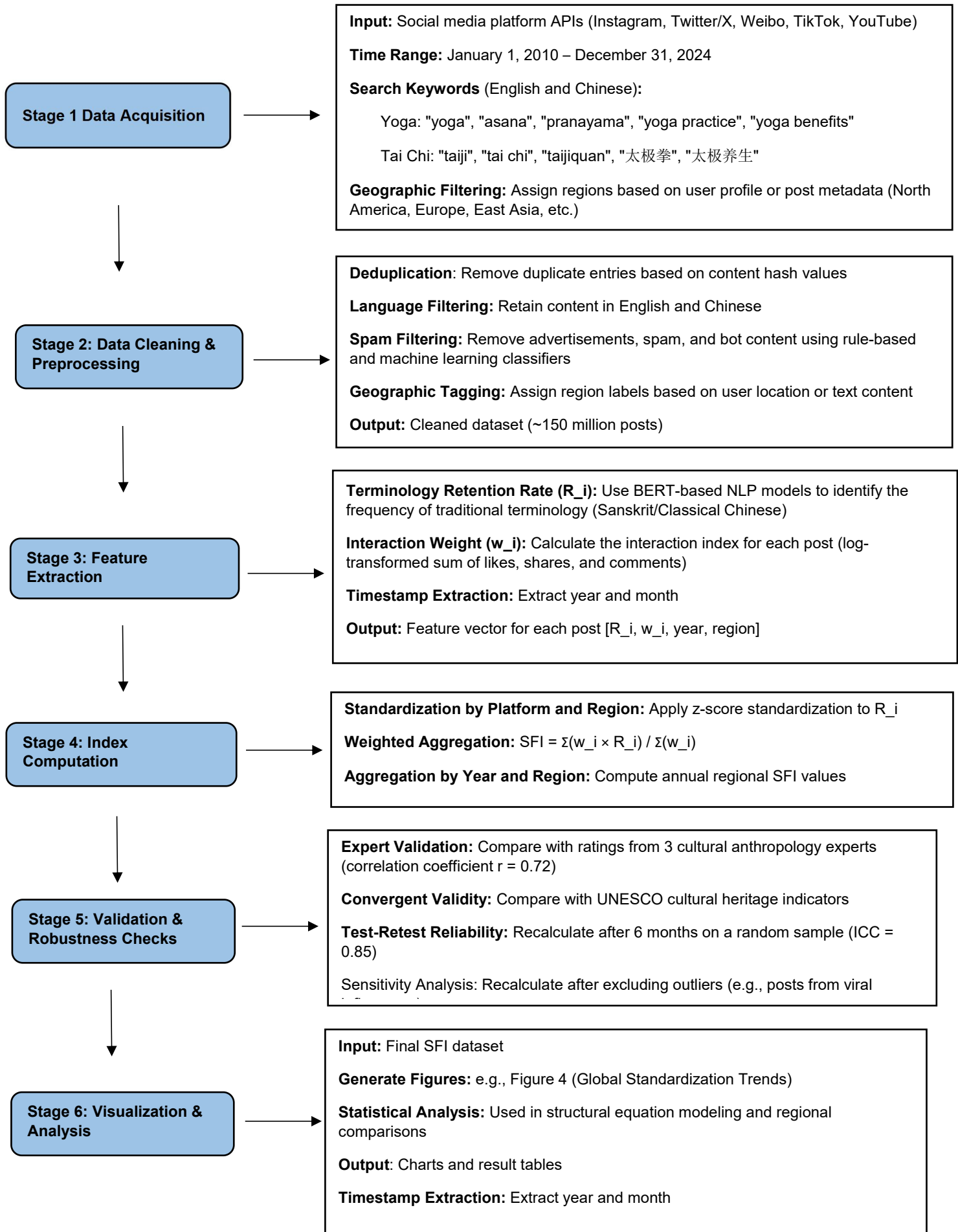

Supplement: Supplementary file 3 [file Image_1.pdf]

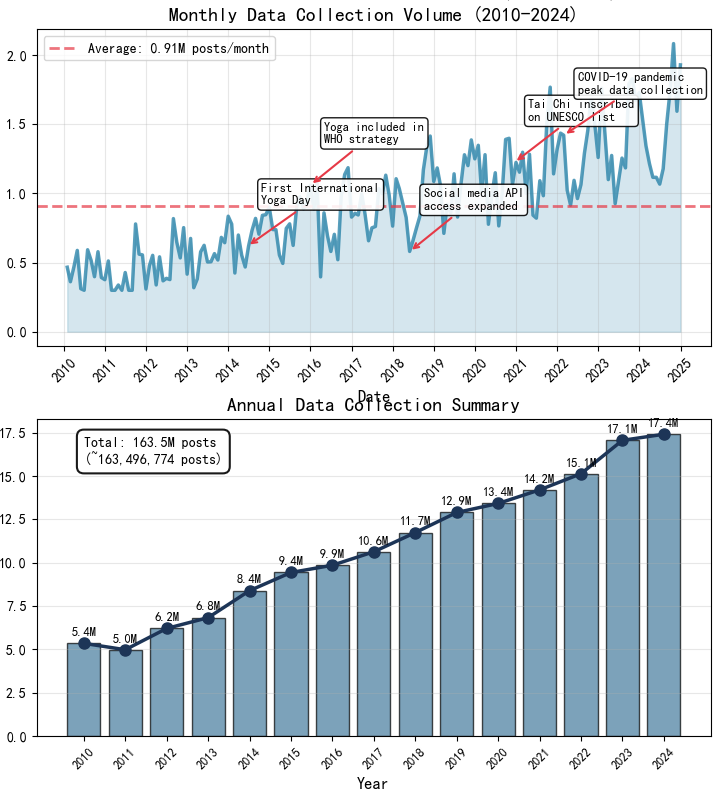

Supplement: Supplementary file 4 [file Image_2.png]

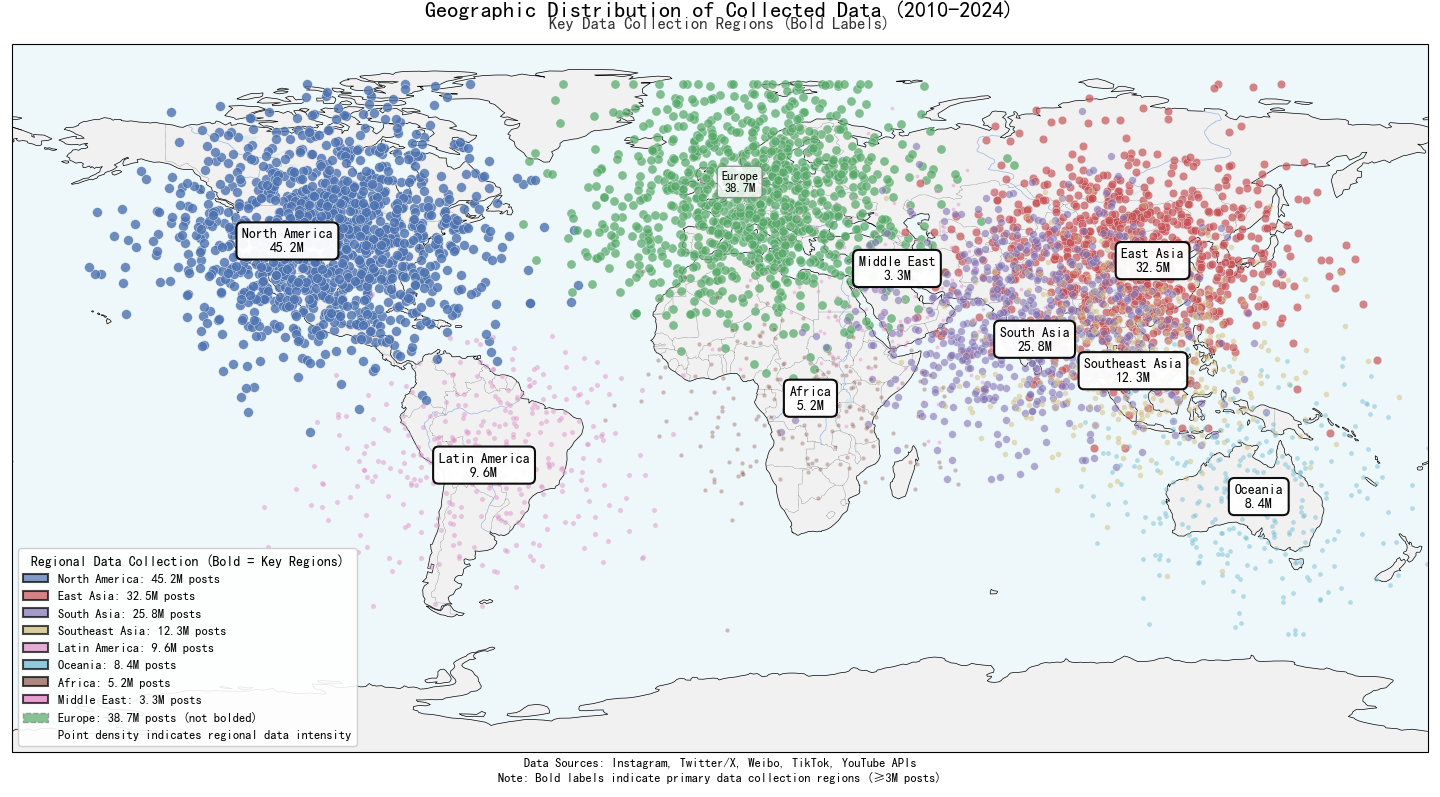

Supplement: Supplementary file 5 [file Image_3.png]
